# Initial Treatment for Acute Low Back Pain and Risk of Opioid Use Disorder and Overdose

**DOI:** 10.1001/jamanetworkopen.2026.39420

**Published:** 2026-10-19

**Authors:** Lisa V. Doan, Anton M. Hung, Mark Olfson, Nicholas T. Williams, Kara E. Rudolph

**Affiliations:** 1 Department of Anesthesiology, Perioperative Care, and Pain Medicine, New York University Grossman School of Medicine, New York; 2 Department of Epidemiology, Mailman School of Public Health, Columbia University, New York, New York; 3 Department of Psychiatry, Columbia University Medical Center, New York, New York

## Abstract

**Importance:**

Guideline-discordant care for acute low back pain (aLBP) is common and has been associated with adverse outcomes.

**Objectives:**

To estimate the incidence of new opioid use disorder (OUD) or overdose diagnosis among opioid-naive patients with Medicaid coverage initiating treatment for aLBP and to evaluate the association between initial treatment modalities and subsequent risk of OUD or overdose diagnosis.

**Design, Setting, and Participants:**

This retrospective cohort study used data from January 1, 2016, to December 31, 2019, with 13 months of follow-up (1-month exposure assessment followed by 12 months of outcome follow-up) from the Transformed Medicaid Statistical Information System Analytic Files from 25 states that expanded Medicaid under the Patient Protection and Affordable Care Act by 2014. Participants included opioid-naive, adults aged less than 65 years who received pain treatment within 1 month of diagnosis of aLBP. Data were analyzed from March 1, 2025, to July 31, 2026.

**Exposures:**

Initial pharmacologic and nonpharmacologic treatments for aLBP.

**Main Outcomes and Measures:**

OUD or overdose diagnosis, defined using diagnostic codes for opioid abuse, opioid dependence, opioid-involved overdose, or a prescription for medication for treating OUD.

**Results:**

In this cohort of  399 500 Medicaid patients who initiated treatment for newly diagnosed aLBP, the median age was 39 (IQR, 30-50) years, and 237 846 (59.5%) were female. The cumulative incidence of OUD or overdose diagnosis was 1.4% at 7 months and 2.3% at 13 months. Initiating vs not initiating higher-dose opioids (*>*50 mg of morphine equivalent) as part of the initial treatment was associated with the greatest risk, increasing risk of OUD or overdose by 146.6% (95% CI, 121.5%-171.7%). Initiating vs not initiating lower-dose opioids prescribed for more than 7 days or gabapentinoids each increased risk by 105.2% (95% CI, 89.0%-121.4%) and 101.6% (95% CI, 84.9%-118.2%), respectively. Nonpharmacologic, noninterventional therapies were associated with reductions in risk of OUD or overdose diagnosis. Initiating vs not initiating chiropractic care was associated with the largest relative risk reduction, decreasing risk by 32.8% (95% CI, 26.8%-38.9%), and initiating physical therapy was associated with 31.1% decreased risk (95% CI, 22.1%-40.1%).

**Conclusions and Relevance:**

In this cohort study, nonpharmacologic, noninterventional treatment was associated with reductions in risk of OUD or overdose diagnosis while initiating treatment with opioids or gabapentinoids was each associated with higher risks. These results support guideline recommendations favoring nonpharmacologic treatments as first-line therapy for aLBP.

## Key Points

**Question:**

What is the incidence of opioid use disorder (OUD) or overdose diagnoses after initiating treatment for acute low-back pain (aLBP), and what is the comparative risk for different treatment options?

**Findings:**

In this cohort study of 399 500 opioid-naive adults with Medicaid insurance, the cumulative incidence of OUD or overdose diagnosis was 2% at 13 months after initiating treatment for aLBP. Initiating higher-dose opioids (*>*50 mg of morphine equivalent) was associated with the highest risk, whereas chiropractic work was associated with the greatest risk reduction.

**Meaning:**

These findings support guideline recommendations favoring nonpharmacologic treatment for aLBP.

## Teaser

This cohort study estimates the association between initial treatment modality and subsequent risk of new-onset opioid use disorder and opioid overdose diagnoses in an opioid-naive population of adults with Medicaid insurance and newly diagnosed acute low back pain.

## Introduction

Acute low back pain (aLBP) is one of the most common reasons adults seek medical care.^1^ In a 2019 survey of US adults, 39% reported low back pain in the prior 3 months.^2^ Overall, the lifetime prevalence of low back pain is estimated to be 60% to 85%.^3,4^ Guidelines from the American College of Physicians for the management of aLBP recommend nonpharmacologic therapies as first-line treatment and suggest nonsteroidal anti-inflammatory drugs or skeletal muscle relaxants if pharmacologic therapy is considered.^5^ Similarly, the 2022 Centers for Disease Control and Prevention Clinical Practice Guideline for Prescribing Opioids for Pain recommends nonopioid pharmacologic and nonpharmacologic therapies as first-line treatment for acute pain, including aLBP.^6^

Despite these recommendations, guideline-discordant care remains common. In an analysis of 2017 US outpatient administrative claims data, 14% of privately insured visits and 19% of Medicaid visits for aLBP had an associated opioid prescription.^7^ In a separate 2018 Medicaid claims analysis, 12.1% of patients received an opioid prescription as initial aLBP treatment, 57.8% received a nonopioid prescription, and 25.1% received noninvasive, nonpharmacologic treatment.^8^ Guideline-discordant care for aLBP is associated with adverse outcomes, including progression to chronic LBP, increased disability, and higher health care costs.^9,10^ In addition, use of higher-risk medications may increase the likelihood of opioid-related harms. Among Medicare patients with aLBP, those who were prescribed opioids and/or gabapentinoids had an almost 2-fold increased odds of overdose-related hospitalization within 90 days of aLBP diagnosis.^11^ Among Medicaid patients with chronic pain, opioids prescribed with gabapentinoids or benzodiazepines were significantly associated with increased risk of opioid use disorder (OUD), while physical therapy was associated with decreased risk.^12^ However, to our knowledge, no prior studies have evaluated the association between treatment modalities for aLBP and OUD outcomes among patients with Medicaid coverage.

Given the high prevalence of aLBP, it is important to understand how initial treatment decisions influence subsequent risk of new-onset diagnoses of OUD and overdose for at least 2 reasons. First, quantifying risks associated with guideline-discordant therapies could reinforce initiatives to promote guideline-concordant care. Second, important differences in risk may exist across the range of pharmacologic and nonpharmacologic therapies for aLBP, including nonopioid treatments that may be perceived as safe options. For example, emerging evidence suggests substantial risks of opioid-related adverse outcomes associated with gabapentinoid prescriptions, even in the absence of an overlapping opioid prescription.^13,14^

In this study, we examined an opioid-naive, adult Medicaid-insured population with newly diagnosed aLBP and estimated the association between initial treatment modality and subsequent risk of new-onset OUD and opioid overdose diagnoses. We hypothesized that nonpharmacologic treatments would be associated with lower risk of OUD and overdose diagnoses, while use of medications such as opioids and gabapentinoids would be associated with higher risk.

## Methods

### Study Sample

Data for this cohort study came from the Transformed Medicaid Statistical Information System (T-MSIS) Analytic Files, including the Demographic and Eligibility, Other Services, Inpatient, and Pharmacy from January 1, 2016, to December 31, 2019. We included data from the 25 states that adopted the Patient Protection and Affordable Care Act Medicaid expansion by 2014 (Arkansas, Arizona, California, Colorado, Connecticut, Delaware, Hawaii, Iowa, Illinois, Kentucky, Massachusetts, Missouri, Minnesota, North Dakota, New Hampshire, New Jersey, New Mexico, Nevada, New York, Ohio, Oregon, Rhode Island, Vermont, Washington, and West Virginia),^15^ excluding Maryland due to unreliable diagnosis code data.^16^ Additional details of the data extraction are given in eMethods 1 in Supplement 1. Columbia University Institutional Review Board approved the study and waived the requirement for written informed consent owing to the use of fully deidentified, retrospective administrative claims data. We followed the Strengthening the Reporting of Observational Studies in Epidemiology (STROBE) reporting guideline.

We created a cohort of Medicaid beneficiaries aged 19 to 63 years with newly diagnosed aLBP, defined as an *International Statistical Classification of Diseases, Tenth Revision* (*ICD-10*), diagnosis of low back pain (eTable 1 in Supplement 1) with no prior low-back pain diagnoses during a 6-month washout period.^17,18^ Patients were required to initiate a new pharmacologic or nonpharmacologic pain treatment (meaning that the treatment was not observed in the washout period) within 1 month of diagnosis. The date of first new treatment initiation was designated as day 0 and marked the start of follow-up. We applied exclusion criteria to focus on a cohort of community-dwelling, opioid-naive adults without another co-occurring or recent pain diagnosis (eTable 2 in Supplement 1), who had Medicaid as their primary insurance, and who did not have another condition that would substantially alter pain management considerations, such as cancer or pregnancy. All cohort eligibility criteria were evaluated during the washout period (Figure 1). Additional details are given in eMethods 2 of Supplement 1. As a sensitivity analysis, we also used a 12-month washout period to more completely capture prior OUD, although this then restricts our cohort to those who remained continuously enrolled for this longer period (eFigure 1 in Supplement 1 shows inclusion and exclusion criteria for this sensitivity analysis).

**Figure 1.**
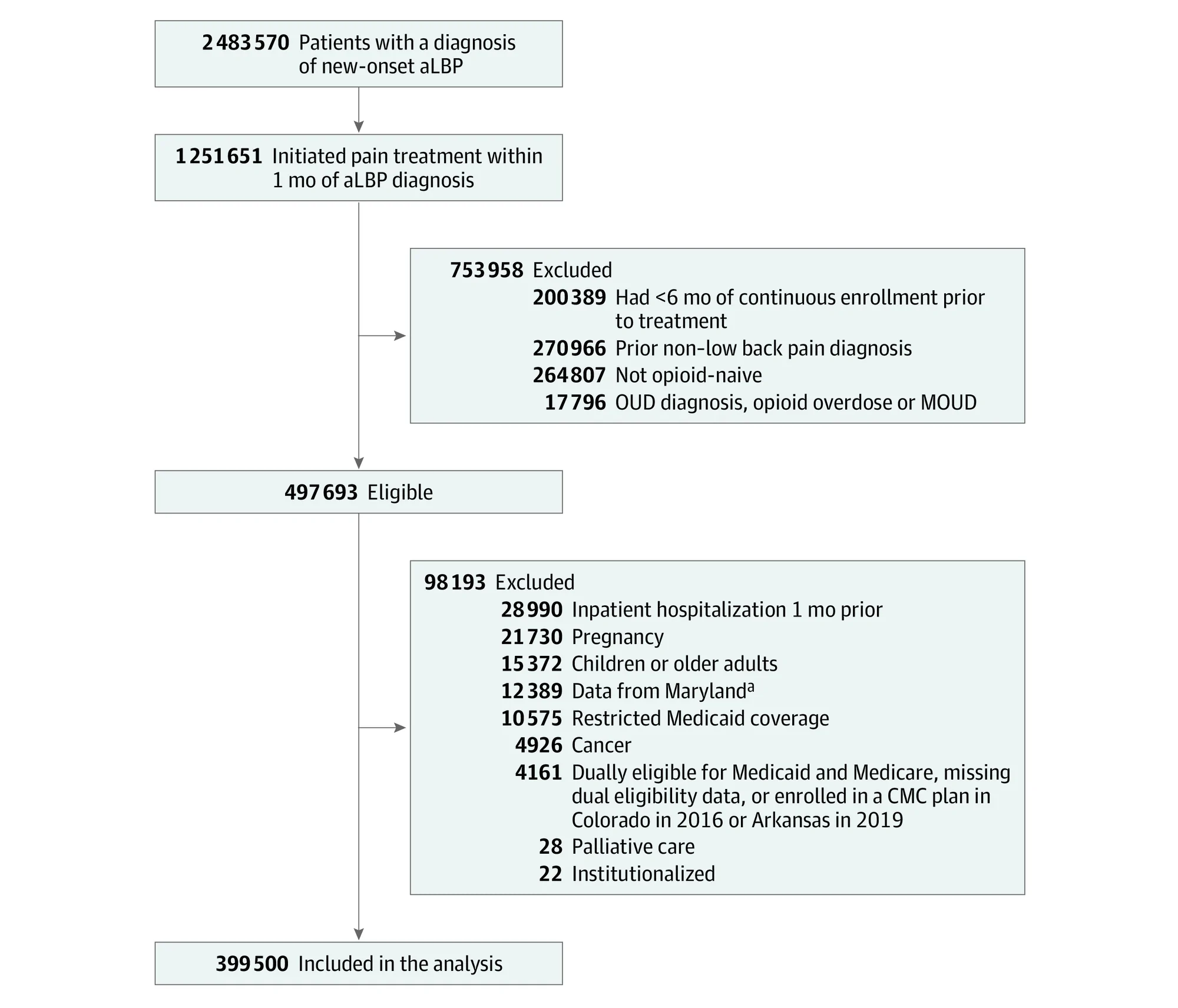
Flowchart of Cohort Inclusion and Exclusion Criteria aLBP indicates acute low back pain; CMC, comprehensive managed care; MOUD, medication for OUD; and OUD, opioid use disorder. ^a^Data were excluded due to unreliable diagnosis code data.

Patients were followed up for 13 months from day 0. Initial treatment was defined during the first 30 days from day 0. Outcomes were assessed after day 30 and through month 13 to ensure temporal separation between exposure and outcome and assessed after 6 months of follow-up (ie, after day 30 through month 7) and after 12 months of follow-up (ie, after day 30 through month 13) for the primary analysis. We chose this period for outcome follow-up to balance (1) enough time to identify a new OUD episode in the claims, yet (2) minimize the proportion of the cohort disenrolled from Medicaid during this period. Individuals were censored at loss of Medicaid enrollment or loss of full-scope or comprehensive benefits (we allowed a 90-day gap and considered loss to have occurred on day 91), Medicare eligibility, death (if recorded), or December 31, 2019 (Figure 2). We conducted 2 sensitivity analyses with alternative definitions of the initial treatment period, as detailed in eMethods 3 in Supplement 1.

**Figure 2.**
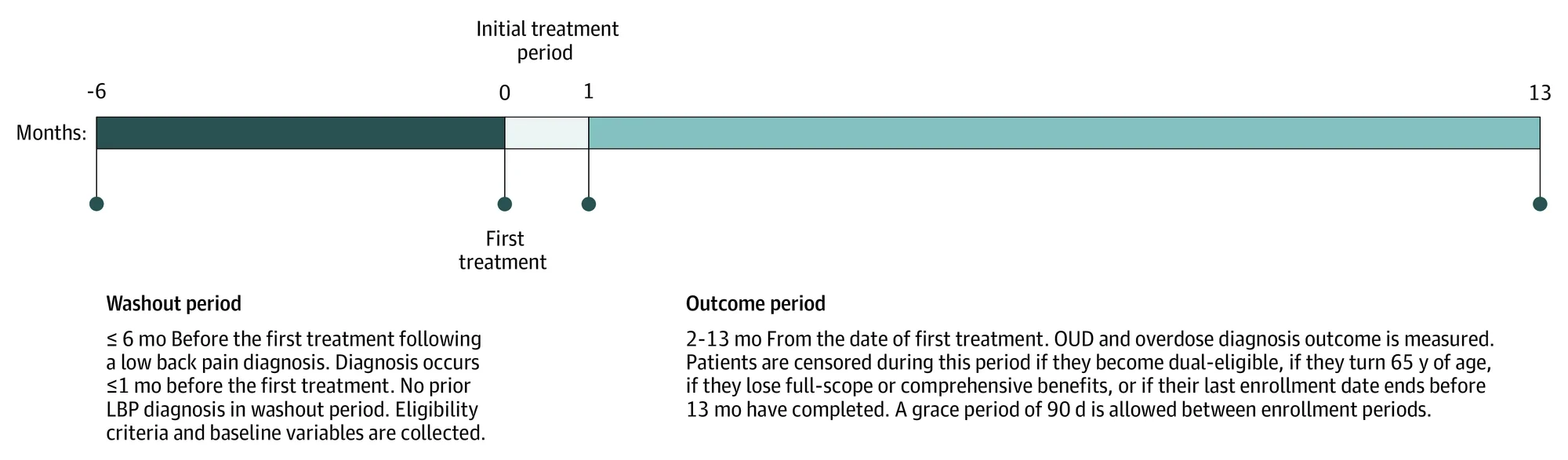
Diagram Showing Study Measure Characterization Timeline The timeline illustrates the assessment windows anchored on the index date of initial acute lower back pain (LBP) treatment (day 0). Eligibility criteria and baseline covariates were evaluated during the 6-month washout period (−182 to −1 days). The outcome follow-up period began 31 days after the index date and extended through month 13, during which incident opioid use disorder (OUD) or overdose and censoring events were captured.

### Covariates

We characterized each patient’s baseline covariates during the 6-month washout period. Demographic covariates included age, sex, race and ethnicity, primary language, marital status, household size, veteran status, income category, and whether or not they received Temporary Assistance for Needy Families benefits or Social Security Disability Insurance benefits. Race and ethnicity were obtained from the T-MSIS Demographic and Eligibility file as administrative demographic variables submitted by state Medicaid programs to the Centers for Medicare & Medicaid Services; categories included American Indian or Alaska Native, Asian, Black, Hispanic, Native Hawaiian or Other Pacific Islander, White, and multiracial. These data were included as possible confounders due to previous literature demonstrating differences in treatment across racial and ethnic groups. Clinical covariates included alcohol use disorder, other substance use disorder, any inpatient or outpatient diagnosis of psychiatric disorders (including bipolar disorder, anxiety disorder, major depressive disorder, attention-deficit/hyperactivity disorder, or other psychiatric disorder), and any mental health counseling. Health system utilization measures included the number of inpatient hospitalizations, outpatient visits, and emergency department visits during the washout period.

### Exposures

The study exposure was the set of new initial treatments received for aLBP. Pharmacologic treatments included acetaminophen, anti-inflammatories, benzodiazepines, gabapentinoids, muscle relaxants, corticosteroids, and opioid analgesics (excluding opioid formulations indicated for OUD treatment).^19,20,21^ Opioid prescriptions were categorized based on days’ supply and maximum daily dosage in milligrams of morphine equivalents (MME), including a supply of 7 days or fewer and a dosage of 50 MME/d or less; a supply of more than 7 days and a dosage of 50 MME/d or less; and a dosage of greater than 50 MME/d. Opioid dosages greater than 50 MME/d are associated with higher risk of overdose compared with lower dosages, with no evidence of clinical benefit for many patients.^6^ Nonpharmacologic treatments included chiropractic therapy, physical therapy, massage therapy, and other interventions, including ablative techniques, botulinum toxin injections, electrical nerve stimulation, intrathecal drug therapies, epidural corticosteroids, and minimally invasive spinal procedures. We additionally defined a category of *other pain management strategy* to encompass less common initial pain treatments that were not analyzed individually. This other pain management strategy category includes acupuncture, topical analgesics, and additional nonopioid analgesics.

### Outcome

Our primary outcome was a new diagnosis of OUD or opioid overdose. We defined these as the presence of an *ICD-10* diagnosis code for OUD,^19,20,22^ an *ICD-10* diagnosis code for nonfatal, unintentional opioid overdose, or receipt of medicine for OUD (methadone or buprenorphine).^23^

### Statistical Analysis

Data were analyzed from March 1, 2025, to July 31, 2026. We estimated the extent to which having vs not having each treatment modality was associated with risk of a diagnosis of new-onset OUD or opioid overdose, leaving other treatments in the set of 14 treatments as observed, and adjusting for measured covariates and censoring. In sensitivity analyses, we estimated a slightly different quantity that is likely to be better supported by the data but has a less intuitive interpretation: the extent to which having each treatment modality vs its observed value was associated with risk of diagnosis of a new-onset OUD or opioid overdose, leaving other treatments in the set of 14 treatments as observed, and adjusting for measured covariates and censoring. For the opioid categories, comparisons were made by setting alternative opioid categories to not received while leaving nonopioid treatments as observed. In eMethods 4 in Supplement 1, we show formal mathematical notation for these estimands and explain the assumptions necessary for these quantities to correspond to causal effects. Relative risks and risk differences were estimated using a doubly robust, nonparametric, targeted minimum loss–based estimator with 2-fold cross-fitting.^24^ Exposure, outcome, and censoring models were estimated using an ensemble^25^ of machine learning algorithms, comprising an intercept-only model, a main-terms generalized linear model, multivariate adaptive regression splines,^26^ gradient boosted machines,^27^ and a least absolute shrinkage and selection operator method.^28^

Analyses were conducted using the lmtp package in R, version 4.4.1 (R Project for Statistical Computing).^29^ All code to replicate analyses is available online.^30^ Estimates with 95% CIs that do not include the null indicate statistical significance at *P* < .05.

## Results

Our analysis included 399 500 Medicaid-insured patients initiating treatment for newly diagnosed aLBP. The median age was 39 (IQR, 30-50) years, of whom 237 846 (59.5%) were female and 161 654 (40.5%) were male. Race and ethnicity data were missing for 55 976 patients (14.0%). Among those with reported race and ethnicity, 6443 (1.9%) were American Indian or Alaska Native, 27 311 (8.0%) were Asian, 62 377 (18.2%) were Black, 72 846 (21.2%) were Hispanic, 2757 (0.8%) were Native Hawaiian or Other Pacific Islander, 171 487 (49.9%) were White, and 303 (0.1%) were multiracial (Table). Of the 224 166 patients who remained uncensored, 27 000 patients (12.0%) developed chronic LBP during the 1-year follow-up,^31^ and 175 344 (43.9%) were right-censored due to Medicaid disenrollment, restricted benefits, death, or dual-eligibility for Medicare.

**Table.** Patient Characteristics for Primary Analysis

Characteristic	No. (%) of patients (N = 399 500)
Baseline covariates (months −6 to 0)	
Age, median (IQR), y	39 (30-50)
Sex	
Male	161 654 (40.5)
Female	237 846 (59.5)
Race and ethnicity	
American Indian or Alaska Native	6443 (1.9)
Asian	27 311 (8.0)
Black	62 377 (18.2)
Hispanic	72 846 (21.2)
Native Hawaiian or Other Pacific Islander	2757 (0.8)
White	171 487 (49.9)
Multiracial	303 (0.1)
No. missing	55 976
Primary language English	319 420 (87.1)
No. missing	32 627
Married or partnered	29 090 (20.7)
No. missing	258 854
Household size	
1	71 585 (63.0)
2	14 687 (12.9)
≥3	27 389 (24.1)
No. missing	285 839
Veteran	665 (0.6)
No. missing	287 918
High income (≥138 FPL)	9114 (2.3)
TANF benefits	39 327 (12.7)
No. missing	90 480
SSDI benefits	
Mandatory or optional	5326 (3.3)
Not applicable	157 725 (96.7)
Missing	236 449
Psychiatric conditions and counseling	
ADHD	5250 (1.3)
Anxiety	45 168 (11.3)
Bipolar disorder	10 870 (2.7)
Depression	35 724 (8.9)
Other mental illness	17 998 (4.5%)
Mental health counseling	26 477 (6.6)
Alcohol use disorder	9455 (2.4)
Other drug use disorder (nonopioid)	28 670 (7.2)
Nicotine	22 607 (5.7)
Cannabis	5249 (1.3)
Sedative	578 (0.1)
Cocaine	2435 (0.6)
Other stimulant	3367 (0.8)
Hallucinogen	183 (0.05)
Inhalant	81 (0.02)
Other psychoactive	3774 (0.9)
Health care utilization	
No. of emergency department visits	
0	300 705 (75.3)
1-2	88 674 (22.2)
≥3	10 121 (2.5)
No. of inpatient hospitalizations	
0	394 694 (98.8)
≥1	4806 (1.2)
No. of outpatient visits	
0	81 107 (20.3)
1-20	307 308 (76.9)
≥21	11 085 (2.8)
Treatments (month 1)	
Acetaminophen	18 103 (4.5)
Anti-inflammatory	193 514 (48.4)
Benzodiazepine	16 765 (4.2)
Chiropractic	40 756 (10.2)
Corticosteroid	60 951 (15.3)
Gabapentinoid	23 228 (5.8)
Other intervention^a^	7627 (1.9)
Muscle relaxant	179 029 (44.8)
Massage therapy	36 239 (9.1)
Physical therapy	65 305 (16.3)
Opioid	83 855 (21.0%)
≤7 d, ≤50 MME/d	54 595 (13.7)
>7 d, ≤50 MME/d	19 672 (4.9)
>50 MME/d	9588 (2.4)
Other treatment	6457 (1.6)
Outcomes (months 2-13)	
OUD or overdose by 7 mo	4317 (1.4)
OUD or overdose by 13 mo	5084 (2.3)
Censoring	
Uncensored through 7 mo	302 139 (75.6)
Uncensored through 13 mo	224 166 (56.1)

Abbreviations: ADHD, attention-deficit/hyperactivity disorder; FPL, federal poverty level; MME, milligrams of morphine equivalent; OUD, opioid use disorder; SSDI, Social Security Disability Insurance; TANF, Temporary Assistance for Needy Families.

^a^ Includes ablative techniques, botulinum toxin injections, electrical nerve stimulation, intrathecal drug therapies, epidural corticosteroids, and minimally invasive spinal procedures.

Anti-inflammatory drugs (193 514 [48.4%]) and muscle relaxants (179 029 [44.8%]) were the most commonly prescribed initial treatments. Physical therapy was the most common nonpharmacologic treatment (65 305 [16.3%]), followed by chiropractic care (40 756 [10.2%]). Among patients who filled an opioid prescription (83 855 [21.0%]), most received prescriptions for a maximum of 50 MME/d or less and 7 or fewer days of supply (54 595 [65.1%] of opioid recipients), while 9588 (11.4%) received a dosage of more than 50 MME/d. In terms of guideline-discordant treatments, such as opioid and gabapentinoid prescriptions, most were initiated within a few days of day 0. For example, of all 91 652 cohort members receiving any opioid prescription within the first 3 months of treatment initiation, 73 302 (80.0% [18.3% of the total cohort]) started the opioid prescription within the first week of treatment (eFigure 2 in Supplement 1).

The cumulative incidence of OUD or overdose diagnosis was 4317 of 302 139 (1.4%) at 7 months and 5084 of 224 166 (2.3%) at 13 months. During months 2 to 13 of follow-up (Figure 3A), opioid prescription dosages greater than 50 MME/d were associated with the largest increase in risk of new-onset diagnosis of OUD or overdose, increasing risk by 146.6% (a 2.5-fold increased risk [95% Wald-type 2-sided CI, 121.5%-171.7%). Lower-dosage opioids (*≤*50 MME/d) prescribed for more than 7 days and gabapentinoids were each associated with a 2.0-fold increased risk (increased risk by 105.2% [95% CI, 89.0%-121.4%] and 101.6% [95% CI, 84.9%-118.2%], respectively). By contrast, opioid dosages of 50 MME/d or less for 7 days or less were associated with a much smaller increase in risk (31.2% [95% CI, 21.7%-40.8%]). Conversely, all nonpharmacologic, noninterventional therapies were associated with reductions in risk of new-onset diagnosis of OUD or overdose. Chiropractic care was associated with the largest relative risk reduction (32.8% [95% CI, 26.8%-38.9%] decrease), followed by physical therapy (31.1% [95% CI, 22.1%-40.1%] decrease). Estimated associations were similar when considering 6 months of outcome follow-up instead of 12 months, although in this case, gabapentinoid prescriptions were associated with more risk than opioid prescription dosages of 50 MME/d or less and more than 7 days of supply (Figure 3B). eTable 4 in Supplement 1 gives all estimates and 95% CIs in terms of both relative risks and risk differences.

**Figure 3.**
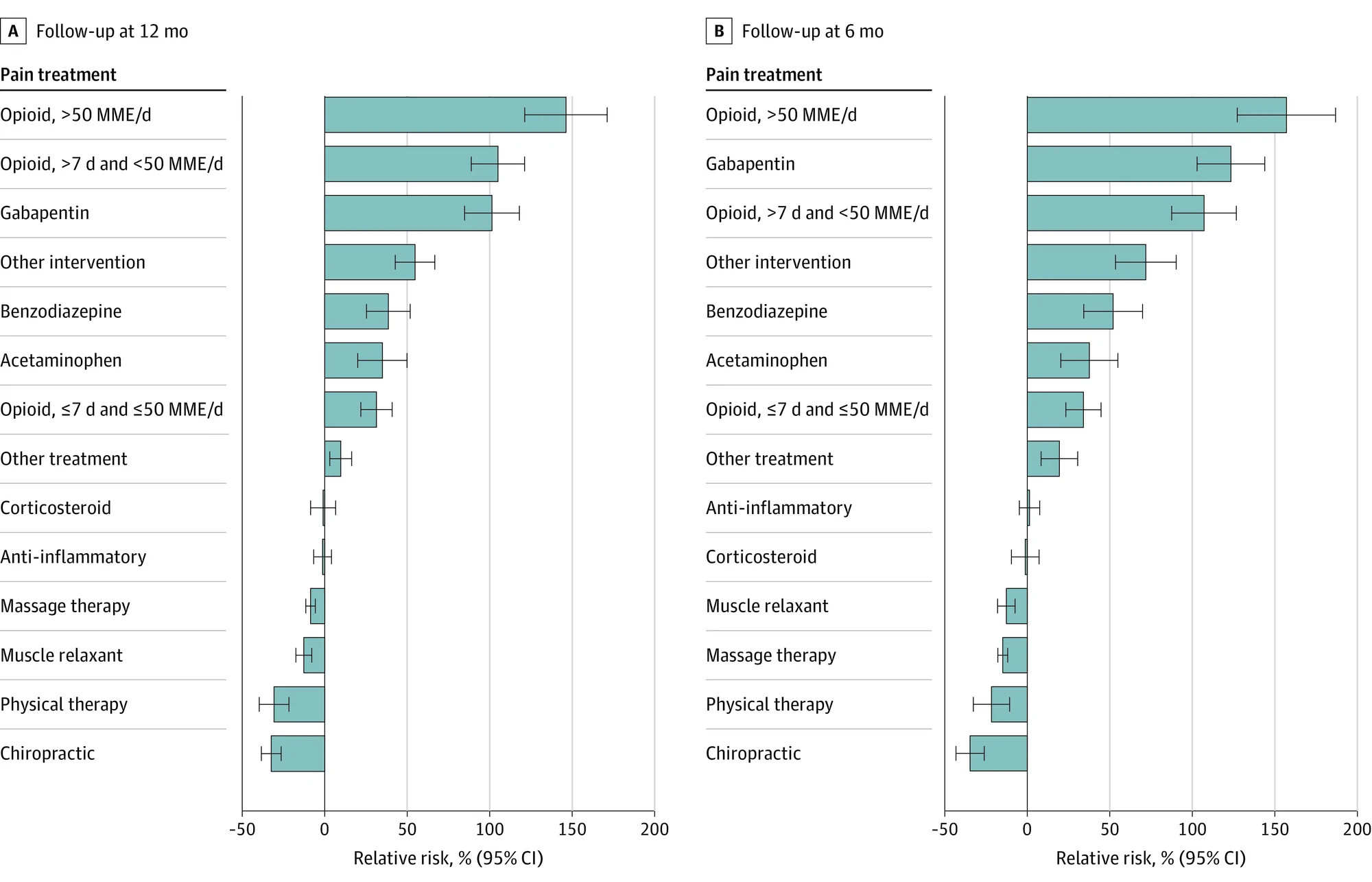
Bar Graphs Showing Estimated Associations of Pain Treatment With Risk of New-Onset Opioid Use Disorder (OUD) or Overdose Graphs show the percentage increase or decrease in risk of OUD or overdose at 12 months (A) and 6 months (B) of follow-up among patients with low back pain and no previous OUD hypothetically adding a treatment to initial treatment combination and no disenrollment vs removing the treatment from initial treatment combination and no disenrollment. Error bars denote 95% CIs. MME indicates milligrams of morphine equivalent.

The frequency of coprescribing may affect the mean risks we estimate, with the coprescription of gabapentinoids and opioids being particularly relevant. eTable 3 in Supplement 1 shows the frequency of their coprescription: gabapentinoids were prescribed to 18 874 patients (4.7%; >4 times more likely to be prescribed) without an overlapping opioid in the initial treatment period, and opioids were prescribed to 79 501 patients (19.9%; >18 times more likely to be prescribed) without an overlapping gabapentinoid.

We conducted several sensitivity analyses, detailed in the Methods, the results of which we show and discuss in eMethods 5, eTables 5 to 7, and eFigures 3 to 10 in Supplement 1. Relative to the results of the primary analysis, the direction and ranking of relative risks in the sensitivity analyses remained consistent, although gabapentinoids conferred the second-greatest risk increase for some results.

## Discussion

In this cohort study of adult, opioid-naive Medicaid-insured patients who initiated treatment for a new episode of aLBP, the cumulative incidence of new-onset diagnosis of OUD or opioid overdose was 1.4% at 7 months and 2.3% at 13 months. Initial treatment patterns were predominantly pharmacologic, most commonly anti-inflammatories, skeletal muscle relaxants, and opioids. Opioids prescribed at a dosage of 50 MME/d or greater or for longer than 7 days and gabapentinoids were each associated with higher risk for a diagnosis of OUD or overdose. By contrast, nonpharmacologic, noninterventional treatment modalities were associated with risk reductions.

For context, in a study using the Truven MarketScan database of commercial claims, the investigators estimated a 0.3% incidence of OUD or overdose in 2016 in adults aged less than 65 years.^32^ We previously estimated a 0.9% 1-year incidence of OUD or overdose among opioid-naive, adult patients insured by Medicaid filling a perioperative opioid prescription.^33^ In the present study, the estimated incidence was higher. This could be because (1) Medicaid-insured patients have higher rates of developing OUD than those with commercial insurance, and (2) compared with populations undergoing surgery, patients with aLBP may experience recurrent episodes of aLBP or transition to chronic LBP, and care for aLBP may be more fragmented.

Consistent with prior studies, guideline-discordant care for aLBP was common. American College of Physicians guidelines for aLBP recommend nonpharmacologic therapies as first-line treatment^5^; however, less than 50% of patients in this cohort received a nonpharmacologic therapy as part of initial care. Although anti-inflammatory medications and skeletal muscle relaxants, the recommended first-line pharmacologic options, were frequently prescribed, opioids were included in 21.0% of initial treatment episodes and 18.3% of cohort patients received an opioid prescription within the first week of treatment.

The 2022 Centers for Disease Control and Prevention Clinical Practice Guidelines state that opioid therapy for acute pain should only be considered when benefits are anticipated to outweigh risks.^6^ A 2023 randomized clinical trial^34^ cast doubt on the potential benefits of opioid therapy for aLBP or acute neck pain. That trial compared guideline-recommended care plus an opioid vs a placebo and found no difference in terms of pain severity at 6 weeks, a small but significant difference in pain severity at 1 year in favor of placebo, and either no differences or small differences in favor of placebo for physical function, quality of life, and global perceived effect.^34^ In our study, even lower-dose, shorter-duration opioid prescriptions were associated with a small increased risk of OUD or overdose diagnosis, with much higher risks associated with opioid dosages greater than 50 MME/d and/or duration of 7 days or longer, corroborating prior work.^12,35,36^

Gabapentinoids were also associated with a higher risk of OUD or overdose diagnoses. Although sometimes perceived as safer alternatives to opioids, emerging evidence suggests important safety concerns. Among Medicare patients with aLBP, gabapentinoid prescribing has been associated with higher risk of drug-related overdose hospitalization within 90 days of aLBP diagnosis.^11^ Use of gabapentinoids in combination with opioids in other populations with acute and chronic pain has been associated with respiratory depression and other opioid-related adverse events, including overdose, OUD, and death.^12,37,38^ Evidence supporting gabapentinoids for LBP is limited, with a systematic review and meta-analysis finding that gabapentin did not provide significant pain improvement compared with placebo, pregabalin was less effective than other active comparators, and gabapentinoids increased the risk of adverse effects.^39^ Accordingly, guidelines for treatment of low back pain from the Department of Veterans Affairs and Department of Defense and guidelines from the National Institute for Health and Care Excellence do not recommend gabapentinoids for low back pain.^40^ In our cohort, gabapentinoids were included in approximately 6% of initial treatment episodes.

All nonpharmacologic and noninterventional therapies were associated with reductions in the risk of overdose or OUD diagnosis, with chiropractic care demonstrating the largest relative risk reduction, followed by physical therapy. This is consistent with prior research that chiropractic care may be associated with decreased odds of receiving opioid therapy for spinal pain^41^ and lower risk of long-term opioid use and OUD.^42^ Likewise, prior research demonstrated associations of early physical therapy with reduced opioid use for treatment of aLBP and lower risk of long-term opioid use among patients with musculoskeletal pain,^43,44^ and lower risk of OUD among Medicaid patients with chronic pain.^12^ In our cohort, chiropractic care was included in only 10.2% and physical therapy in 16.3% of initial treatment episodes.

### Limitations

This study has some limitations. As an observational analysis of administrative claims data, results may be affected by residual confounding, misclassification, underreporting of diagnoses, and unmeasured differences in pain severity and functional status. In terms of the outcome, although use of *ICD-10* codes for opioid abuse or dependence (F11.1X and F11.2X, respectively) in Medicaid claims has a sensitivity of nearly 90%, specificity is likely lower, and misclassification is a concern.^45,46,47^ For this reason, we refer to the outcome as OUD or overdose diagnosis. Over-the-counter medications and prescriptions covered by self-pay were not included (although self-pay is less common in the population with Medicaid coverage), and details on actual medication consumption, including illicit use, are unknown. The analysis did not account for all comorbidities or subtypes of aLBP that may influence treatment choices. Physician-directed exercise was also not captured. It has been estimated that 2% to 48% of patients with aLBP transition to chronic low back pain, and some patients may have recurrent episodes of aLBP.^48^ Defining chronic LBP as claims for LBP greater than 90 days apart,^19,31^ we estimated that 12.1% of our study’s aLBP cohort transitioned to chronic LBP, but the trajectory of aLBP resolution or progression was unknown. Finally, our findings may not be generalizable beyond the Medicaid-insured adult population.

## Conclusions

In this cohort study of Medicaid-insured adults with newly diagnosed aLBP, initial treatment decisions were associated with meaningful downstream consequences. Pharmacologic therapies, especially higher-dose or longer-duration opioid prescriptions and gabapentinoids, were associated with increased risk of subsequent diagnosis of OUD or overdose. In contrast, nonpharmacologic, noninterventional therapies, chiropractic care, and physical therapy in particular were associated with reductions in risk. These findings support prioritizing guideline-recommended nonpharmacologic, noninterventional therapies as first-line care for aLBP. Pharmacologic therapies can be considered after weighing potential benefits against longer-term risks. When opioids are prescribed, minimizing dose and duration remain prudent.^6,7^

## Notes

Supplement 1. **eMethods 1.** Additional Detail on Extracting TAF Data **eMethods 2.** Additional Detail on Exclusion Criteria **eMethods 3.** Additional Detail on Sensitivity Analyses **eMethods 4.** Additional Detail on Statistical Analysis **eTable 1.** *ICD-10* Codes Used for Assembling a Cohort of Individuals Diagnosed With Low Back Pain **eTable 2.** *ICD-10-CM* Code Prefixes to Identify Non–Low-Back Pain Conditions **eFigure 1.** Sensitivity Analysis Redefining the Washout Period to Begin 12 Months Before the Initial Treatment Date **eFigure 2.** Days Until Opioid Start Among Those With an Opioid Treatment in First 90 Days (n = 91 652) **eTable 3.** Frequency of Gabapentinoid and Opioid Exposure Categories Overlap Defined by Duration and Opioid Dose (n = 399 500) **eMethods 5.** Sensitivity Analysis Results **eTable 4.** Patients With Low Back Pain and No Previous OUD Receiving a Pain Treatment Within 1 Month of Diagnosis **eFigure 3.** Estimated Relative Risk of Incident OUD Over 6 Months of Follow-Up Among Patients With Low Back Pain and No Previous OUD Receiving a Pain Treatment Within 1 Month of Diagnosis **eFigure 4.** Estimated Relative Risk of Incident OUD Over 12 Months of Follow-Up Among Patients With Low Back Pain and No Previous OUD Receiving a Pain Treatment Within 1 Month of Diagnosis **eTable 5.** Descriptive Table for Sensitivity Analysis With Initial Treatment Period as Ending at the First 30-Day Gap in Pain Treatment, or at the End of 3 Months **eFigure 5.** Estimated Relative Risk of Incident OUD Over 6 Months of Follow-Up: Sensitivity Analysis Redefining the Initial Treatment Period as Ending at the First 30-Day Gap in Pain Treatment or at the End of 3 Months **eFigure 6.** Estimated Relative Risk of Incident OUD Over 12 Months of Follow-Up: Sensitivity Analysis Redefining the Initial Treatment Period as Ending at the First 30-Day Gap in Pain Treatment, or at the End of 3 Months **eTable 6.** Descriptive Table for Sensitivity Analysis: Initial Treatment Period as Ending at the First 7-Day Gap in Pain Treatment, or at the End of 3 Months **eFigure 7.** Estimated Relative Risk of Incident OUD Over 6 Months of Follow-Up: Sensitivity Analysis Redefining the Initial Treatment Period as Ending at the First 7-Day Gap in Pain Treatment, or at the End of 3 Months **eFigure 8.** Estimated Relative Risk of Incident OUD Over 12 Months of Follow-Up: Sensitivity Analysis Redefining the Initial Treatment Period as Ending at the First 7-Day Gap in Pain Treatment, or at the End of 3 Months **eTable 7.** Descriptive Table for Sensitivity Analysis. Washout Period Adjusted to be 12 Months (n = 259 724) **eFigure 9.** Estimated Relative Risk of Incident OUD Over 6 Months of Follow-Up: Sensitivity Analysis Redefining the Washout Period to Begin 12 Months Before the Initial Treatment Date **eFigure 10.** Estimated Relative Risk of Incident OUD Over 12 Months of Follow-Up: Sensitivity Analysis Redefining the Washout Period to Begin 12 Months Before the Initial Treatment Date
eReferences

Supplement 2.
Data Sharing Statement
