# Supplementary material for: Initial Treatment for Acute Low Back Pain and Risk of Opioid Use Disorder and Overdose
Source: JAMA Netw Open. 2026 Oct 19;9(10):e2639420. doi: 10.1001/jamanetworkopen.2026.39420 (PMC13681206; doi:10.1001/jamanetworkopen.2026.39420)
Supplement: Supplementary file 1 [file jamanetwopen-e2639420-s001.pdf]

Supplementary Online Content

Doan LV, Hung AM, Olfson M, Williams NT, Rudolph KE. Initial treatment for acute low back pain and risk of opioid use disorder and overdose. *JAMA Netw Open.* 2026;9(10):e2639420. doi:10.1001/jamanetworkopen.2026.39420

eMethods 1. Additional Detail on Extracting TAF Data

eMethods 2. Additional Detail on Exclusion Criteria

eMethods 3. Additional Detail on Sensitivity Analyses

eMethods 4. Additional Detail on Statistical Analysis

eTable 1. *ICD-10* Codes Used for Assembling a Cohort of Individuals Diagnosed With Low Back Pain

eTable 2. *ICD-10-CM* Code Prefixes to Identify Non–Low-Back Pain Conditions

eFigure 1. Sensitivity Analysis Redefining the Washout Period to Begin 12 Months Before the Initial Treatment Date

eFigure 2. Days Until Opioid Start Among Those With an Opioid Treatment in First 90 Days (n = 91 652)

eTable 3. Frequency of Gabapentinoid and Opioid Exposure Categories Overlap Defined by Duration and Opioid Dose (n = 399 500)

eMethods 5. Sensitivity Analysis Results

eTable 4. Patients With Low Back Pain and No Previous OUD Receiving a Pain Treatment Within 1 Month of Diagnosis

eFigure 3. Estimated Relative Risk of Incident OUD Over 6 Months of Follow-Up Among Patients With Low Back Pain and No Previous OUD Receiving a Pain Treatment Within 1 Month of Diagnosis

eFigure 4. Estimated Relative Risk of Incident OUD Over 12 Months of Follow-Up Among Patients With Low Back Pain and No Previous OUD Receiving a Pain Treatment Within 1 Month of Diagnosis

eTable 5. Descriptive Table for Sensitivity Analysis With Initial Treatment Period as Ending at the First 30-Day Gap in Pain Treatment, or at the End of 3 Months

eFigure 5. Estimated Relative Risk of Incident OUD Over 6 Months of Follow-Up: Sensitivity Analysis Redefining the Initial Treatment Period as Ending at the First 30-Day Gap in Pain Treatment or at the End of 3 Months

eFigure 6. Estimated Relative Risk of Incident OUD Over 12 Months of Follow-Up: Sensitivity Analysis Redefining the Initial Treatment Period as Ending at the First 30-Day Gap in Pain Treatment, or at the End of 3 Months

eTable 6. Descriptive Table for Sensitivity Analysis: Initial Treatment Period as Ending at the First 7-Day Gap in Pain Treatment, or at the End of 3 Months

eFigure 7. Estimated Relative Risk of Incident OUD Over 6 Months of Follow-Up: Sensitivity Analysis Redefining the Initial Treatment Period as Ending at the First 7-Day Gap in Pain Treatment, or at the End of 3 Months

© 2026 Doan LV et al. *JAMA Network Open*.

eFigure 8. Estimated Relative Risk of Incident OUD Over 12 Months of Follow-Up: Sensitivity Analysis Redefining the Initial Treatment Period as Ending at the First 7-Day Gap in Pain Treatment, or at the End of 3 Months

eTable 7. Descriptive Table for Sensitivity Analysis. Washout Period Adjusted to be 12 Months (n = 259 724)

eFigure 9. Estimated Relative Risk of Incident OUD Over 6 Months of Follow-Up: Sensitivity Analysis Redefining the Washout Period to Begin 12 Months Before the Initial Treatment Date

eFigure 10. Estimated Relative Risk of Incident OUD Over 12 Months of Follow-Up: Sensitivity Analysis Redefining the Washout Period to Begin 12 Months Before the Initial Treatment Date

eReferences

This supplementary material has been provided by the authors to give readers additional information about their work.

© 2026 Doan LV et al. *JAMA Network Open*.

eMethods 1 Additional Detail on Extracting TAF Data

TAF data obtained for this study included the first 24 months of coverage for non-dual-eligible, non-pregnant, adult beneficiaries (ages 19-64), for whom Medicaid is the primary insurer, excluding eligibility group codes 05, 06, 07, 08, 23, 25, 29, 31, 33, 34, 44, 45, 53, 54, 61, 62, 64, 65, 66, and 68. Those with missing eligibility group codes were retained. Dual eligibility was defined as being non-dual-eligible for both Medicaid and Medicare for all enrolled months in the year. The years 2016-2019 and the following states were included: AZ, AR, CA, CO, CT, DE, HI, IA, IL, KY, MD, MA, MI, MN, NJ, NV, NH, NM, NY, ND, OH, OR, RI, VT, WA, WV. Additional subgroups were excluded, namely intellectual disability, blind/vision problem, deaf/hearing problem, and dementia/Alzheimer's. The 2016-2019 TAF Demographic and Eligibility files were searched for beneficiaries who meet all of the above criteria in the same year. The cohort was randomly reduced to 19,999,999 beneficiaries. For those beneficiaries, TAF data were extracted in July 2022 from the Demographic and Eligibility, Other Services, Inpatient, and Pharmacy files for 2016 release 2, 2017 release 2, 2018 release 2, and 2019 release 1.

eMethods 2 Additional Detail on Exclusion Criteria

We excluded patients who were not continuously enrolled in Medicaid during the washout period with full-scope or comprehensive benefits. We excluded patients who, during the washout period, had any history of: OUD diagnosis, overdose, medication for treating OUD (MOUD), opioid prescription, pregnancy, dual eligibility for Medicaid and Medicare, cancer, or institutionalization. We also excluded individuals who were diagnosed with any other pain condition during the 3 months preceding day 0, and excluded those who had their initial pain diagnosis or therapy in an inpatient or residential setting. Because of data completeness concerns for comprehensive managed care enrollees in AR in 2019 and CO in 2016, we excluded beneficiaries enrolled in managed care plans in these state-years.[1]

eMethods 3 Additional Detail on Sensitivity Analyses

In the first sensitivity analysis, we defined the end of the initial treatment period as being at the end of the first 30-day gap in pain treatment (aligned with previous research[2,3]) or at the end of 3 months (to align with the definition of chronic pain[4]), whichever occurred first. In the second sensitivity analysis, we defined the end of the initial treatment period as being at the end of the first 7-day gap in pain treatment (also aligned with previous research[3]) or at the end of 3 months, whichever occurred first. We assessed outcomes during months 4-15 in these two sensitivity analyses to preserve temporality while maintaining 12-months of outcome follow-up.

© 2026 Doan LV et al. *JAMA Network Open*.

eMethods 4 Additional Detail on Statistical Analysis

We assume observed data $\mathbf{O} = (\mathbf{W}, \mathbf{A}, C_1, C_1Y_1, C_2, C_2Y_2)$, where: $\mathbf{W}$ represents the covariates measured during the washout period; $\mathbf{A}$ represents initial pain management treatment variables; C_t represents an indicator of remaining uncensored by follow-up time t, where $t = 1$ corresponds to month 7 (outcome measured during months 2-7) and $t = 2$ corresponds to month 13 (outcome measured during months 2-13); finally, Y_t represents the outcome by time t, which is observed among those who remain uncensored.
We estimated two causal estimands. The first can be denoted

$$E(Y_t^{\mathrm{d}1_i,\bar{C}_t=\bar{1}} - Y_t^{\mathrm{d}0_i,\bar{C}_t=\bar{1}}), \tag{1}$$

where $\mathrm{d}1_i$ is defined as $\mathrm{d}1_i(\mathbf{A}) = (A_i = 1, \mathbf{A} \backslash A_i)$, $\mathrm{d}0_i$ is defined as $\mathrm{d}0_i(\mathbf{A}) = (A_i = 0, \mathbf{A} \backslash A_i)$, we use an overbar to denote variable history such that $\bar{C}_2 = \bar{1}$ denotes remaining uncensored at both follow-up timepoints, and we use the notation $Y^{\mathrm{d},\bar{1}}$ to indicate the counterfactual value of the outcome had the set of treatments been modified according to policy d and had censoring not occurred, possibly contrary to fact. The second causal estimand can be denoted

$$E(Y_t^{\mathrm{d}1_i,\bar{C}_t=\bar{1}} - Y_t^{\bar{C}_t=\bar{1}}), \tag{2}$$

where the second component of the contrast leaves the set of pain treatments as observed. $\mathrm{d}1_i$ and $\mathrm{d}0_i$ are defined as above for $i \in \{1, ..., 7, 12, 13, 14\}$. However, when hypothetically intervening on one of the three opioid variables, the other two variables must necessarily be set to 0. Consequently, for
$i \in \{8, 9, 10\}, \mathrm{d}1_i(\mathbf{A}) = (A_i = 1, \mathbf{A} \in \{A_8, A_9, A_{10}\} \backslash A_i = (0, 0), \mathbf{A} \in \{A_1, ..., A_9, A_{10} \backslash A_i)$,
$\mathrm{d}0_i$ is defined as
$\mathrm{d}0_i(\mathbf{A}) = (A_i = 0, \mathbf{A} \in \{A_8, A_9, A_{10}\} \backslash A_i = (0, 0), \mathbf{A} \in \{A_1, ..., A_7, A_{11}, ..., A_{14}\} \backslash A_i)$.
Each of these causal estimands are equal to the following to statistical estimands, which are a function only of observed data and therefore estimable, under the identification assumptions given next:

$$E[E(Y_t \mid \bar{C}_t = \bar{1}, \mathbf{A} = \mathrm{d}1_i, \mathbf{W}) - E(Y_t \mid \bar{C}_t = \bar{1}, \mathbf{A} = \mathrm{d}0_i, \mathbf{W})], \tag{3}$$

$$E[E(Y_t \mid \bar{C}_t = \bar{1}, \mathbf{A} = \mathrm{d}1_i, \mathbf{W}) - E(Y_t \mid \bar{C}_t = \bar{1}, \mathbf{A}, \mathbf{W})]. \tag{4}$$

The identification assumptions under which the above statistical estimands equal the causal estimands are: 1) conditional exchangeability, meaning that there is no unobserved/unmeasured confounding of the relationship between the set of treatments and outcome conditional on covariates and that there is no unobserved/unmeasured confounding between censoring and the outcome conditional on the covariates and treatment; and 2) positivity, meaning that if it is possible to find a beneficiary with observed data $(\mathbf{a}, \bar{c}, \mathbf{w})$, then it is possible to find a beneficiary with data $(\mathrm{d}1_i(\mathbf{a}), \bar{1}, \mathbf{w})$ and $(\mathrm{d}0_i(\mathbf{a}), \bar{1}, \mathbf{w})$ for the first estimand pair and with data $(\mathrm{d}1_i(\mathbf{a}), \bar{1}, \mathbf{w})$ for the second estimand, for $i \in \{1 : 14\}$. In other words, this states that if it is possible to find a beneficiary with covariates $\mathbf{w}$, censoring $\bar{c}$, treatment set $\mathbf{a}$, it is possible to find a beneficiary with covariates $\mathbf{w}$, who are uncensored, and treatment set $\mathrm{d}1_i(\mathbf{a})$ and $\mathrm{d}0_i(\mathbf{a})$ for $i \in \{1 : 14\}$.

© 2026 Doan LV et al. *JAMA Network Open*.

eTable 1: ICD-10 Codes Used for Assembling a Cohort of Individuals Diagnosed With Low Back Pain

Pain category	ICD-10 code
Arthritis/Joint pain	M4005, M4015, M40205, M40295, M4035, M4036, M4037, M4045, M4046, M4047, M4055, M4056, M4057, M4105, M4106, M4107, M41115, M41116, M41117, M41125, M41126, M41127, M4125, M4126, M4127, M4135, M4145, M4146, M4147, M4155, M4156, M4157, M4205, M4206, M4207, M4215, M4216, M4217, M4305, M4306, M4307, M4315, M4316, M4317, M435X5, M435X6, M435X7, M438X5, M438X6, M438X7, M4625, M4626, M4627, M4635, M4636, M4637, M4845XA, M4846XA, M4847XA, M532X5, M532X6, M9903, M9983, S32000K, S32001K, S32002K, S32008K, S32009K, S32010K, S32011K, S32012K, S32018K, S32019K, S32020K, S32021K, S32022K, S32028K, S32029K, S32030K, S32031K, S32032K, S32038K, S32039K, S32040K, S32041K, S32042K, S32048K, S32049K, S32050K, S32051K, S32052K, S32058K, S32059K, S329XXK
Back Pain	M4325, M4326, M4327, M455, M456, M457, M4605, M4606, M4607, M4645, M4646, M4647, M4655, M4656, M4657, M4685, M4686, M4687, M4695, M4696, M4697, M4715, M4716, M4725, M4726, M4727, M47815, M47816, M47817, M47895, M47896, M47897, M4805, M48061, M4807, M4815, M4816, M4817, M4825, M4826, M4827, M4835, M4836, M4837, M488X5, M488X6, M488X7, M4985, M4986, M4987, M5105, M5106, M5115, M5116, M5117, M5125, M5126, M5127, M5135, M5136, M5137, M5145, M5146, M5147, M5185, M5186, M5187, M519, M532X7, M5385, M5386, M5387, M5405, M5406, M5407, M5415, M5416, M5417, M5430, M5431, M5432, M5440, M5441, M5442, M545, M9923, M9933, M9943, M9953, M9963, M9973
Neurologic Pain	M47015, M47016, G541, G544

© 2026 Doan LV et al. *JAMA Network Open*.

eTable 2: ICD-10-CM Code Prefixes to Identify Non-Low-Back Pain Conditions. Patients with these pre-existing conditions (diagnosed during 3 months prior to initial treatment) were excluded from the study cohort

Pain Category	ICD-10 Prefixes
**Arthritis / Joint / Bone Pain**	A18, A52, D48, E08–E11, E13, M00–M02, M04–M08, M11–M25, M32–M36, M40–M43, M46, M48, M53–M54, M60–M63, M65–M67, M70–M72, M75–M77, M79–M81, M84–M96, M99, Q68, R25, R26, R29, S02, S12, S22, S32, S42, S49, S52, S59, S62, S72, S79, S82, S89, S92, S99
**Back Pain**	M08, M43, M45–M49, M51, M53–M54, M62, M96, M99, Q76
**Back/Neck Pain Unspecified**	M25, M46–M48
**Headache**	G43, G44
**Misc Pain**	F45, G89, K58, M26, N30, N41, N94, R10, R52
**Neck Pain**	M43, M46–M48, M50, M53–M54, M99
**Neurologic Pain**	A52, B02, E08–E11, E13, G50, G52, G54–G58, G60–G65, G90, G99

Note: 3-character prefixes map to multiple clinical categories (e.g., M54), specific 4th and 5th character sub-codes dictated final category assignment. A complete CSV listing all individual codes is available in the online repository.

eFigure 1: Sensitivity Analysis Redefining the Washout Period to Begin 12 Months Before the Initial Treatment Date. Flowchart of cohort inclusion and exclusion criteria.

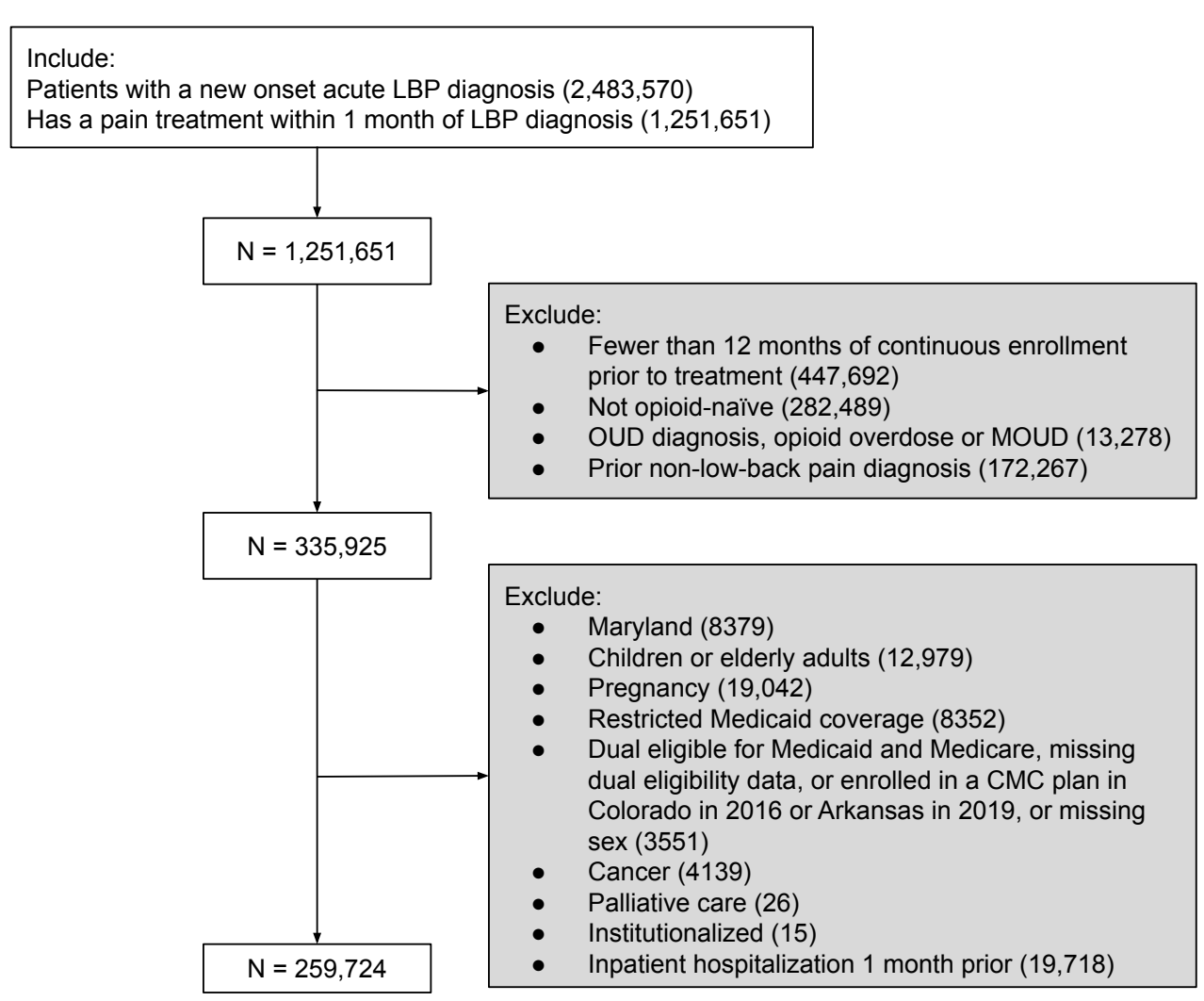

© 2026 Doan LV et al. *JAMA Network Open*.

eFigure 2: Days Until Opioid Start Among Those With an Opioid Treatment in First 90 days (n=91,652). 73,302 individuals received opioids in first 7 days.

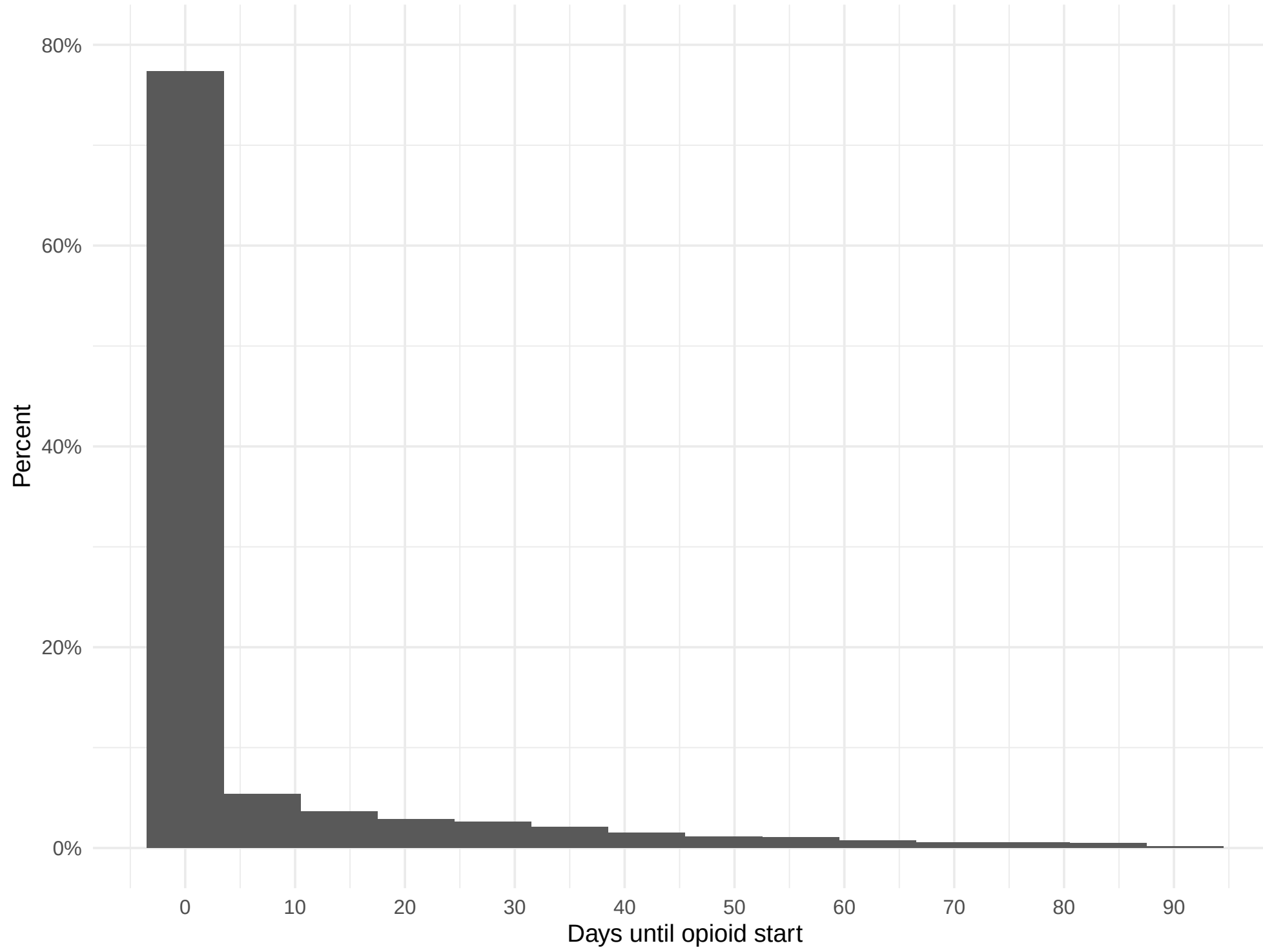

eTable 3: Frequency of Gabapentinoid and Opioid Exposure Categories Overlap Defined by Duration and Opioid Dose (N = 399,500).

Co-prescription category	Number (%)
gabapentinoid *with* overlapping opioid $\leq$ 7 days, $\leq$ 50 MME	1697 (0.4%)
gabapentinoid *with* overlapping opioid > 50 MME	781 (0.2%)
gabapentinoid *with* overlapping opioid > 7 days, $\leq$ 50 MME	1876 (0.5%)
gabapentinoid *without* overlapping opioid	18874 (4.7%)
opioid $\leq$ 7 days, $\leq$ 50 MME *without* overlapping gabapentinoid	52898 (13.2%)
opioid > 50 MME *without* overlapping gabapentinoid	8807 (2.2%)
opioid > 7 days, $\leq$ 50 MME *without* overlapping gabapentinoid	17796 (4.5%)

eMethods 5 Sensitivity Analysis Results

We conducted several sensitivity analyses, detailed in the Methods.

© 2026 Doan LV et al. *JAMA Network Open*.

Figures 3 and 4 show results for the sensitivity analysis where we considered an alternative estimand that quantifies the relative risks of new-onset diagnosis of OUD or overdose for each pain treatment versus its observed value. Relative to the primary analysis' with-versus-without-treatment comparison, the direction and ranking of relative risks remained largely consistent, although gabapentin conferred the second-greatest risk increase over 12 months of outcome follow-up. Effect sizes were attenuated, as expected. Most notably, lower dose, shorter duration opioid prescriptions resulted in minimal change in risk (14.6%, 95% CI: 7.7%, 21.5%).

Inferences were also robust in the two additional sensitivity analyses that redefined the initial treatment episode as specified in the Methods section, using 30-day and 7-day allowable gaps between treatments for up to month 3 (descriptive statistics shown in Tables 5-6 and association estimates shown in Figures 5 - 8) and in the final sensitivity analysis that used a 12-month washout period (descriptive statistics shown in Table 7 and association estimates shown in Figures 9-10).

eTable 4: Patients With Low Back Pain and No Previous OUD Receiving a Pain Treatment Within 1 Month of Diagnosis. Estimated relative risks and risk differences of incident OUD hypothetically adding a treatment to initial treatment combination and no disenrollment versus removing the treatment from initial treatment combination and no disenrollment.

Treatment	6-month RD (95% CI)	6-month RR (95% CI)	12-month RD (95% CI)	12-month RR (95% CI)
Acetaminophen	0.005 (0.003, 0.008)	1.38 (1.20, 1.55)	0.007 (0.004, 0.011)	1.35 (1.20, 1.50)
Anti-inflammatory	0.000 (-0.001, 0.001)	1.01 (0.95, 1.07)	-0.000 (-0.002, 0.001)	0.98 (0.93, 1.04)
Benzodiazepine	0.007 (0.005, 0.010)	1.52 (1.34, 1.70)	0.008 (0.005, 0.011)	1.38 (1.25, 1.52)
Chiropractic	-0.005 (-0.006, -0.004)	0.65 (0.57, 0.74)	-0.007 (-0.009, -0.006)	0.67 (0.61, 0.73)
Gabapentin	0.016 (0.014, 0.019)	2.23 (2.03, 2.44)	0.021 (0.018, 0.024)	2.02 (1.85, 2.18)
Intervention	0.010 (0.007, 0.012)	1.72 (1.53, 1.90)	0.012 (0.009, 0.014)	1.55 (1.43, 1.67)
Muscle relaxant	-0.002 (-0.003, -0.001)	0.87 (0.82, 0.93)	-0.003 (-0.004, -0.002)	0.87 (0.82, 0.92)
Massage therapy	-0.002 (-0.003, -0.002)	0.85 (0.82, 0.88)	-0.002 (-0.003, -0.001)	0.91 (0.88, 0.94)
Physical therapy	-0.003 (-0.005, -0.002)	0.78 (0.67, 0.89)	-0.007 (-0.009, -0.005)	0.69 (0.60, 0.78)
Steroid	-0.000 (-0.001, 0.001)	0.99 (0.90, 1.07)	-0.000 (-0.002, 0.001)	0.99 (0.91, 1.06)
Opioid $\leq$ 7days $\leq$ 50mme	0.004 (0.003, 0.005)	1.34 (1.23, 1.45)	0.006 (0.004, 0.008)	1.31 (1.22, 1.41)
Opioid $>$ 7days $\leq$ 50mme	0.013 (0.011, 0.015)	2.07 (1.88, 2.27)	0.020 (0.017, 0.023)	2.05 (1.89, 2.21)
Opioid $>$ 50mme	0.019 (0.016, 0.022)	2.57 (2.27, 2.87)	0.028 (0.023, 0.033)	2.47 (2.22, 2.72)
Other treatment	0.003 (0.001, 0.004)	1.19 (1.08, 1.31)	0.002 (0.001, 0.003)	1.09 (1.03, 1.16)

© 2026 Doan LV et al. *JAMA Network Open*.

eFigure 3: Estimated Relative Risk of Incident OUD Over 6 Months of Follow-Up Among Patients with Low Back Pain and No Previous OUD Receiving a Pain Treatment Within 1 Month of Diagnosis. Analysis hypothetically adding a treatment to initial treatment combination and no disenrollment, versus holding treatments as observed and no disenrollment. Error bars denote the 95% CI of the estimate.

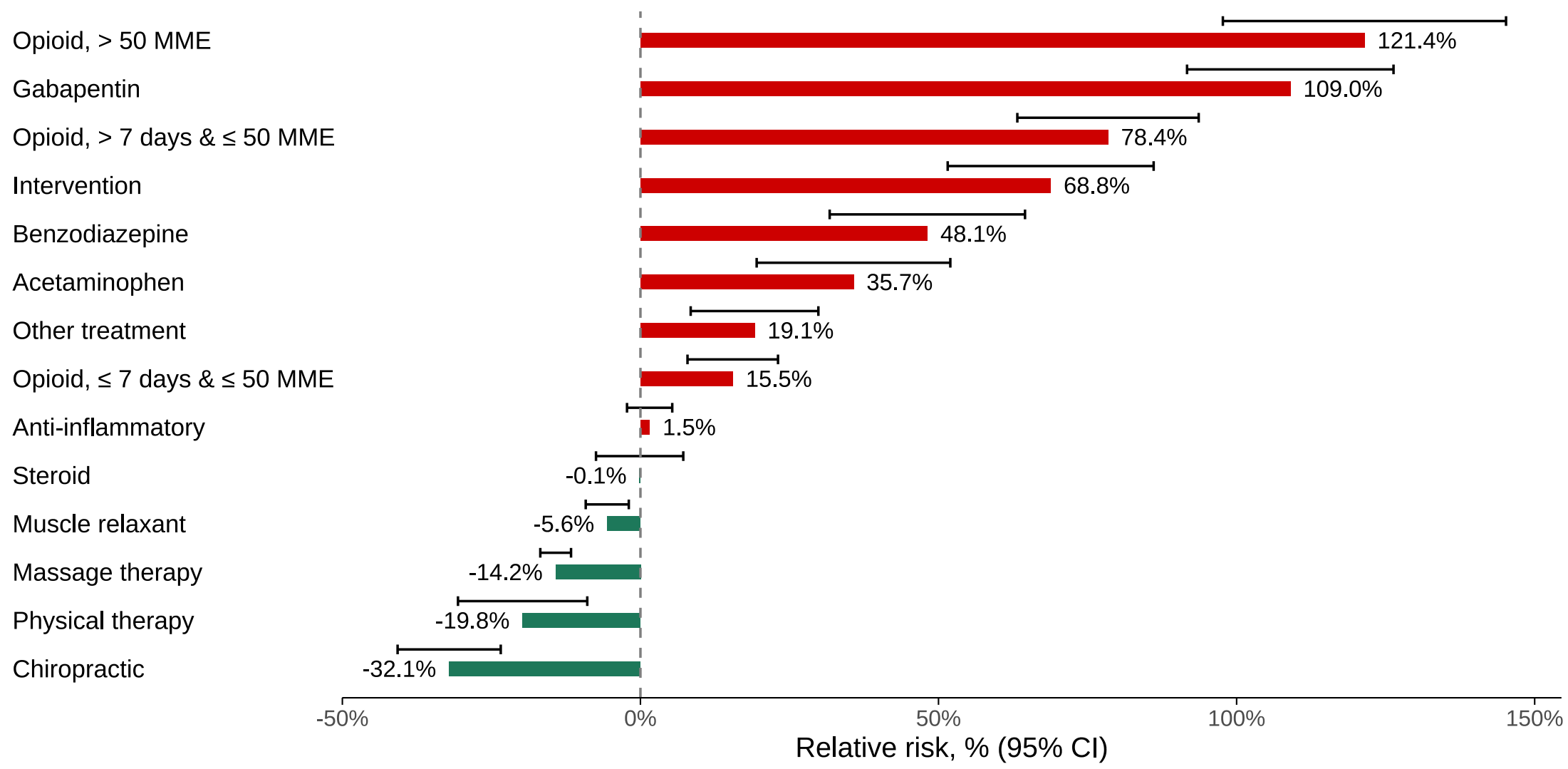

eFigure 4: Estimated Relative Risk of Incident OUD Over 12 Months of Follow-Up Among Patients with Low Back Pain and No Previous OUD Receiving a Pain Treatment Within 1 Month of Diagnosis. Analysis under hypothetically adding a treatment to initial treatment combination and no disenrollment, versus holding treatments as observed and no disenrollment. Error bars denote the 95% CI of the estimate.

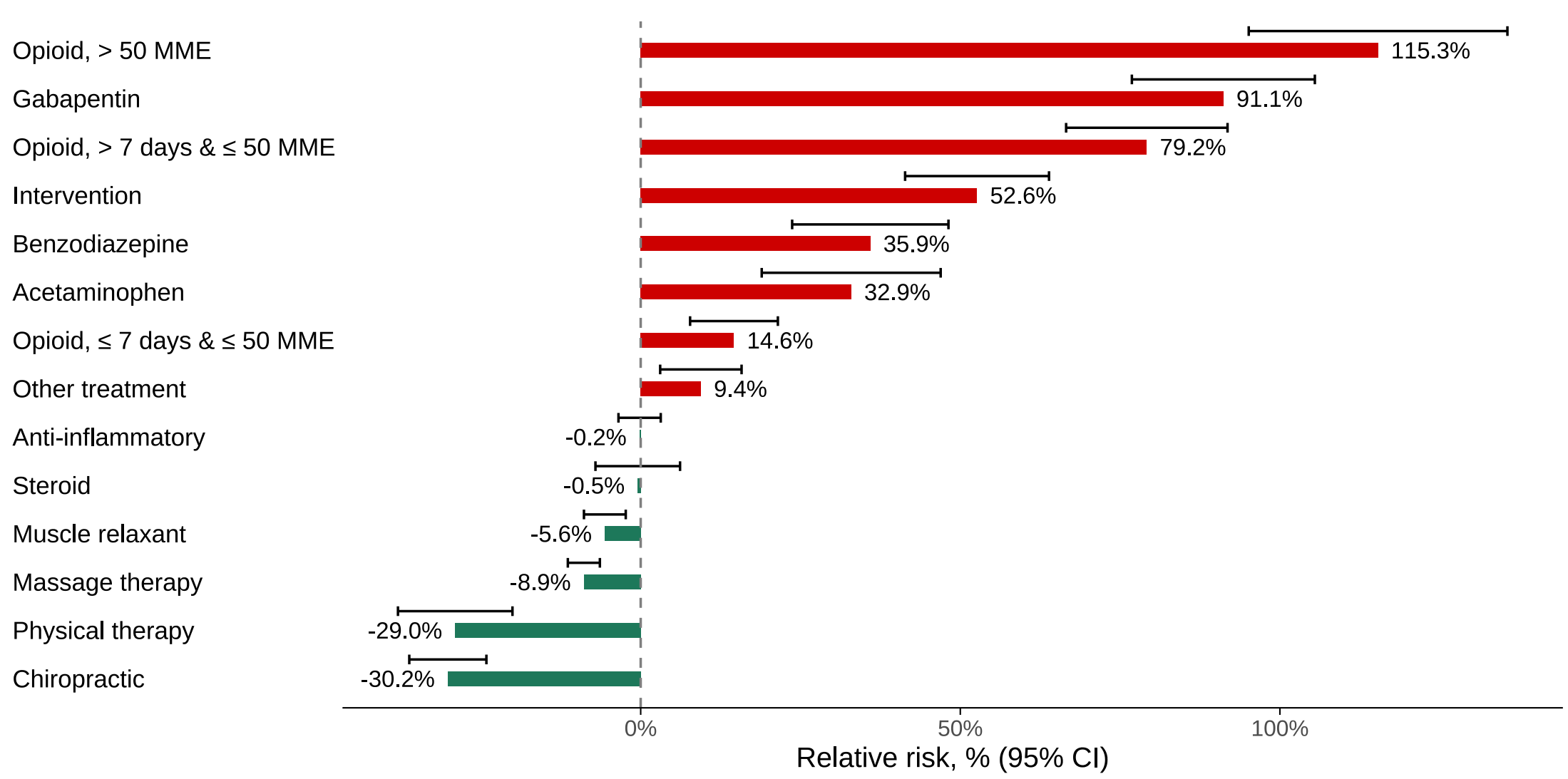

© 2026 Doan LV et al. *JAMA Network Open*.

eTable 5: Descriptive Table for Sensitivity Analysis With Initial Treatment Period as Ending at the First 30-Day Gap in Pain Treatment, or at the End of 3 Months

Characteristic	N=399,500
**Treatments (months 1-3)**	
Acetaminophen	20280 (5.1%)
Anti-inflammatory	198640 (49.7%)
Benzodiazepine	19821 (5.0%)
Chiropractic	41758 (10.5%)
Gabapentin	26938 (6.7%)
Intervention	10964 (2.7%)
Muscle relaxant	183108 (45.8%)
Massage therapy	43171 (10.8%)
Physical therapy	75292 (18.8%)
Steroid	67161 (16.8%)
Opioid	91652 (22.9%)
$\leq$ 7 days, $\leq$ 50 MME	55943 (14.0%)
$>$ 7 days, $\leq$ 50 MME	23590 (5.9%)
$>$ 50 MME	12119 (3.0%)
Other treatment	7513 (1.9%)
**Outcomes (months 4-15)**	
OUD or overdose by 9 months	4744 (1.7%)
OUD or overdose by 15 months	5126 (2.5%)
**Censoring**	
Uncensored through 9 months	277172 (69.4%)
Uncensored through 15 months	202650 (50.7%)

Binary values formatted as N (%).

© 2026 Doan LV et al. *JAMA Network Open*.

eFigure 5: Estimated Relative Risk of Incident OUD Over 6 Months of Follow-up: Sensitivity Analysis Redefining the Initial Treatment Period as Ending at the First 30-Day Gap in Pain Treatment or at the End of 3 Months. Analysis under hypothetically adding a treatment to initial treatment combination and no disenrollment, versus removing the treatment from initial treatment combination and no disenrollment. Error bars denote the 95% CI of the estimate.

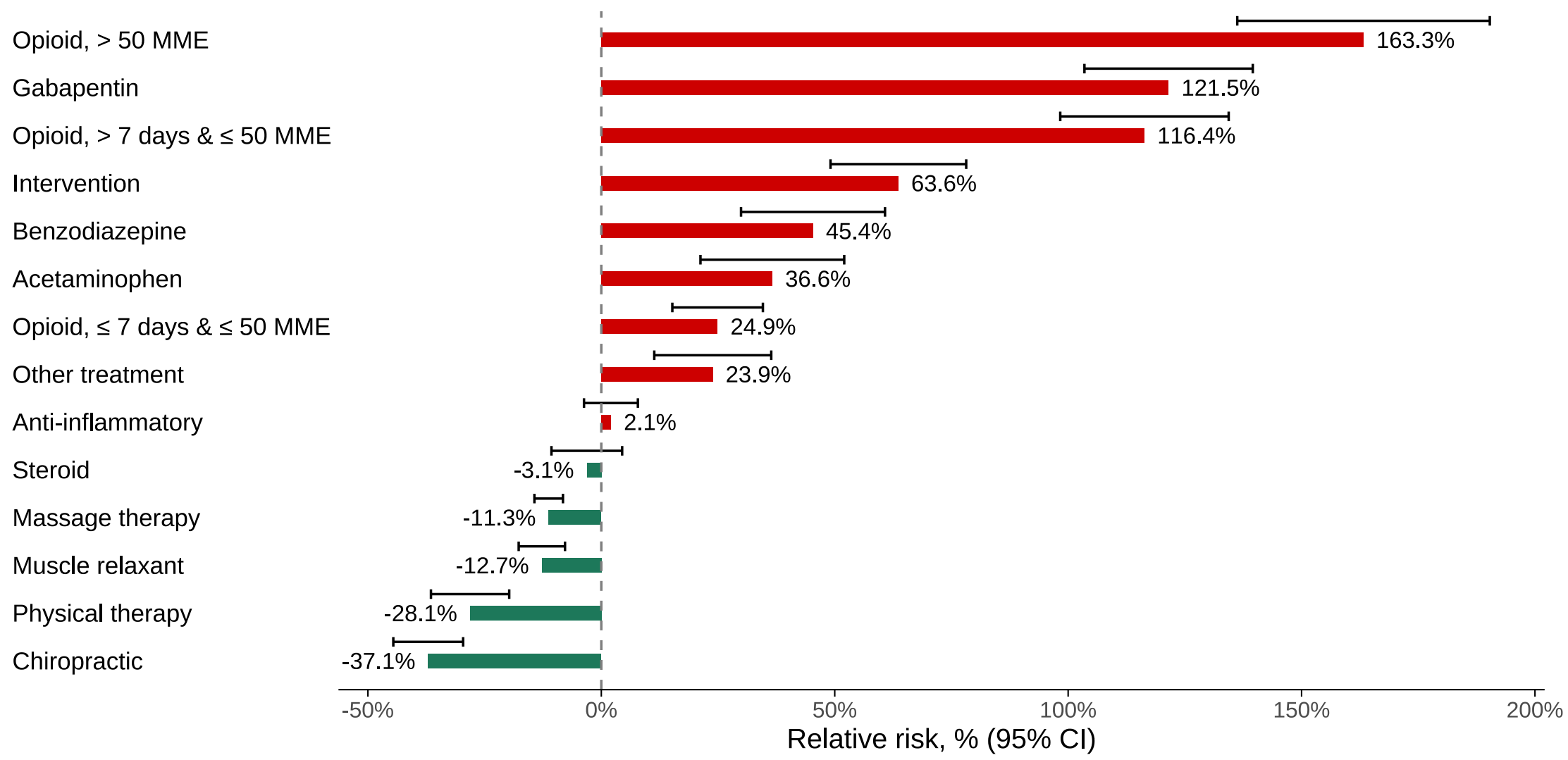

© 2026 Doan LV et al. *JAMA Network Open*.

eFigure 6: Estimated Relative Risk of Incident OUD Over 12 Months of Follow-up: Sensitivity Analysis Redefining the Initial Treatment Period as Ending at the First 30-Day Gap in Pain Treatment or at the End of 3 Months. Analysis under hypothetically adding a treatment to initial treatment combination and no disenrollment, versus removing the treatment from initial treatment combination and no disenrollment. Error bars denote the 95% CI of the estimate.

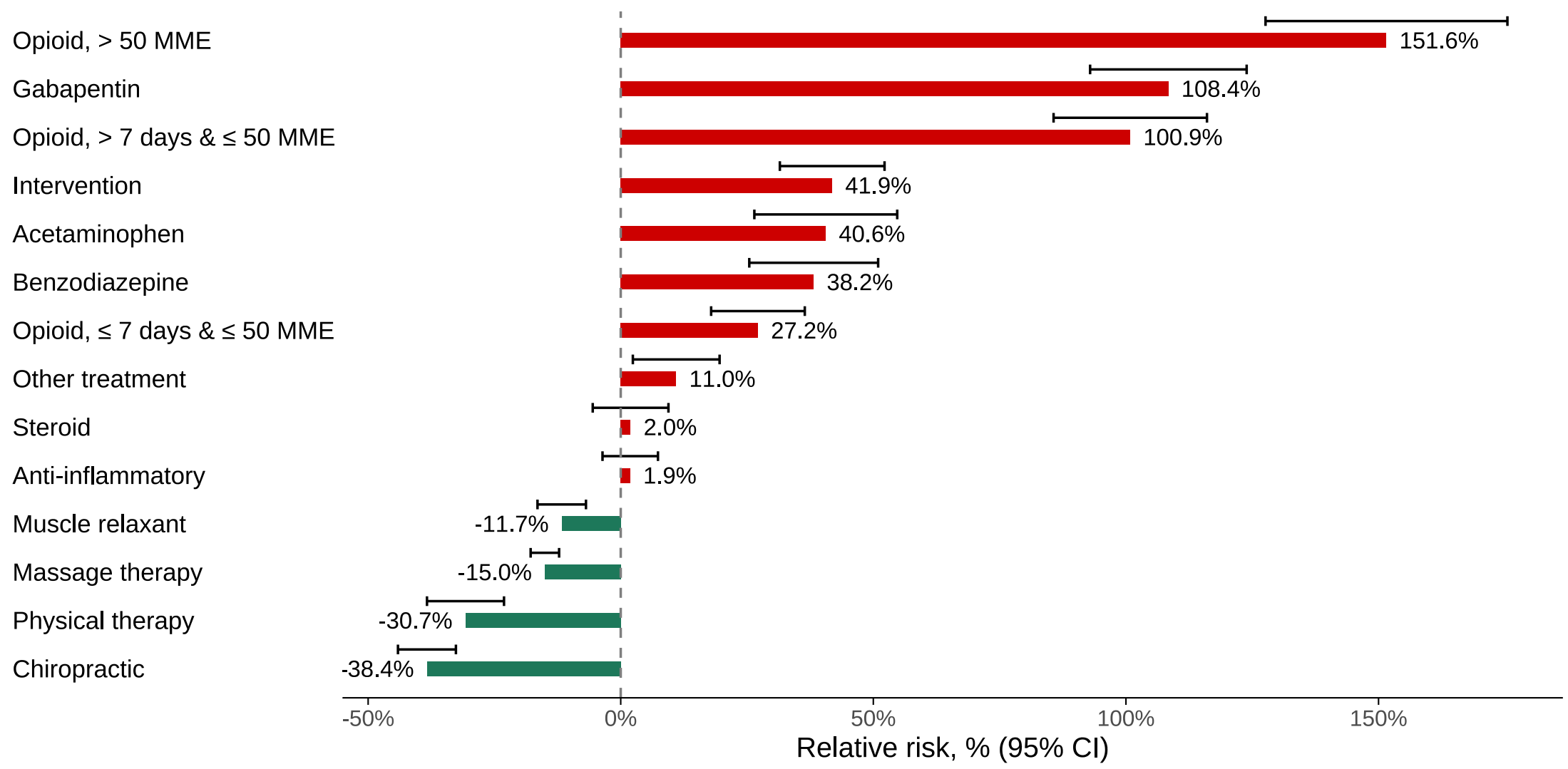

© 2026 Doan LV et al. *JAMA Network Open*.

eTable 6: Descriptive Table for Sensitivity Analysis: Initial Treatment Period as Ending at the First 7-Day Gap in Pain Treatment, or at the End of 3 Months

Characteristic	N=399,500
**Treatments (months 1-3)**	
Acetaminophen	17805 (4.5%)
Anti-inflammatory	191289 (47.9%)
Benzodiazepine	16651 (4.2%)
Chiropractic	40589 (10.2%)
Gabapentin	23165 (5.8%)
Intervention	8536 (2.1%)
Muscle relaxant	177731 (44.5%)
Massage therapy	35466 (8.9%)
Physical therapy	65422 (16.4%)
Steroid	60315 (15.1%)
Opioid	83069 (20.8%)
$\leq$ 7 days, $\leq$ 50 MME	53575 (13.4%)
$>$ 7 days, $\leq$ 50 MME	19510 (4.9%)
$>$ 50 MME	9984 (2.5%)
Other treatment	6402 (1.6%)
**Outcomes (months 4-15)**	
OUD or overdose by 9 months	4744 (1.7%)
OUD or overdose by 15 months	5126 (2.5%)
**Censoring**	
Uncensored through 9 months	277172 (69.4%)
Uncensored through 15 months	202650 (50.7%)

Binary values formatted as N (%).

© 2026 Doan LV et al. *JAMA Network Open*.

eFigure 7: Estimated Relative Risk of Incident OUD Over 6 Months of Follow-up: Sensitivity Analysis Redefining the Initial Treatment Period as Ending at the First 7-Day Gap in Pain Treatment, or at the End of 3 Months. Analysis under hypothetically adding a treatment to initial treatment combination and no disenrollment, versus removing the treatment from initial treatment combination and no disenrollment. Error bars denote the 95% CI of the estimate.

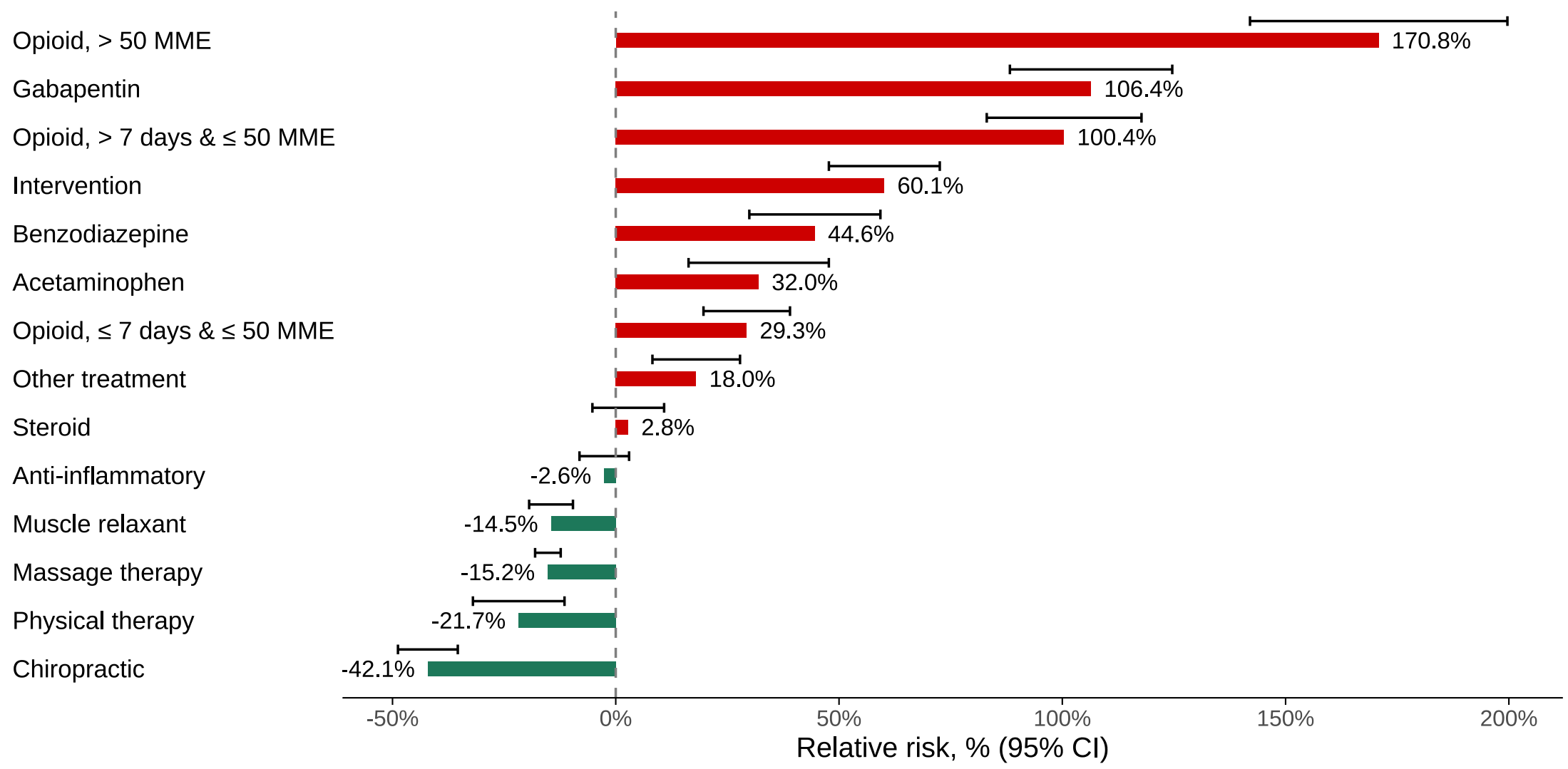

© 2026 Doan LV et al. *JAMA Network Open*.

eFigure 8: Estimated Relative Risk of Incident OUD Over 12 Months of Follow-up: Sensitivity Analysis Redefining the Initial Treatment Period as Ending at the First 7-Day Gap in Pain Treatment, or at the End of 3 Months. Analysis under hypothetically adding a treatment to initial treatment combination and no disenrollment, versus removing the treatment from initial treatment combination and no disenrollment. Error bars denote the 95% CI of the estimate.

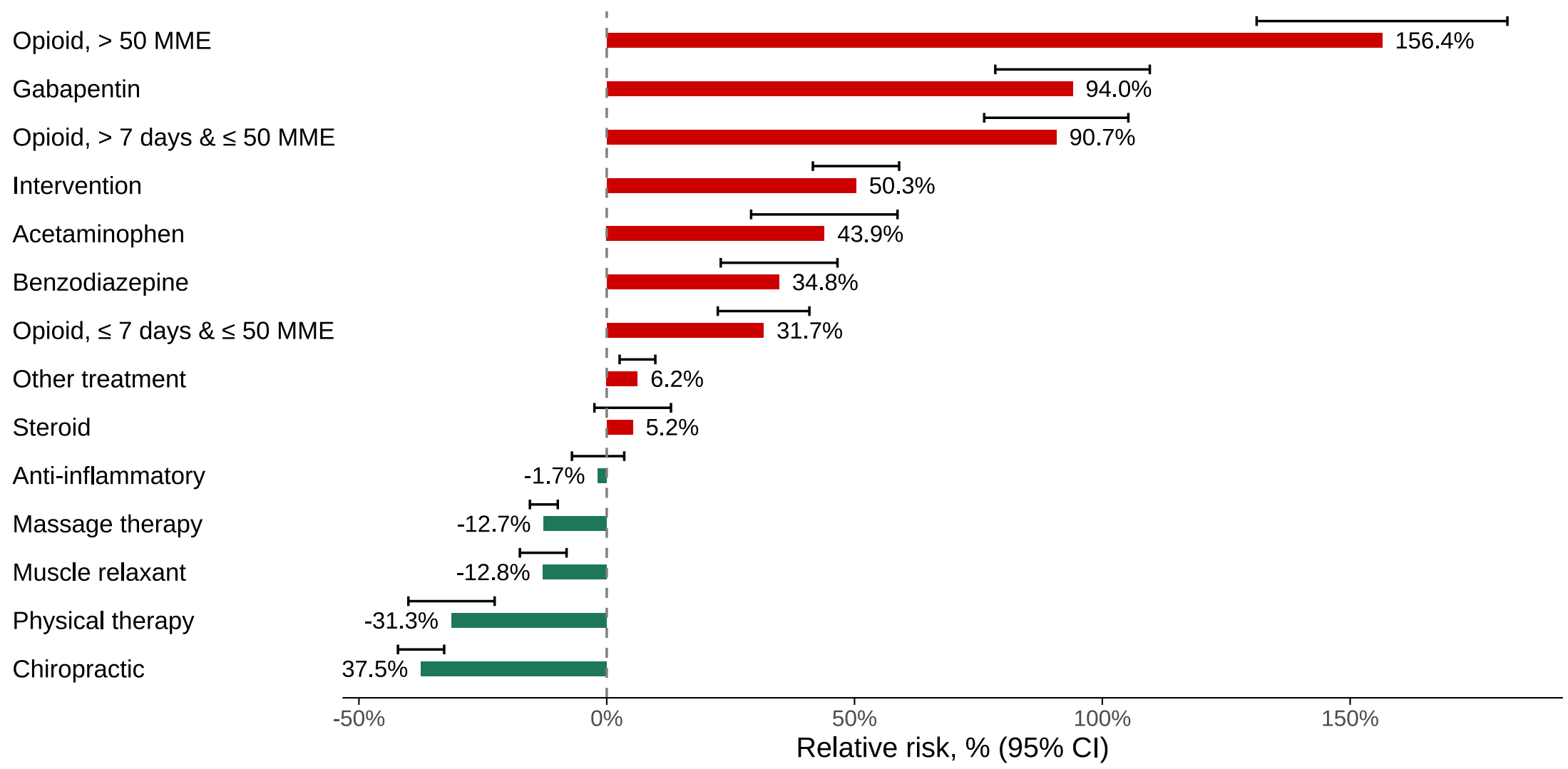

© 2026 Doan LV et al. *JAMA Network Open*.

eTable 7: Descriptive Table for Sensitivity Analysis. Washout Period Adjusted to be 12 Months (N=259,724).

Characteristic	Count (%)
**Baseline covariates (months -6 to 0)**	
Age, median (IQR)	39 (30, 50)
Sex	
Male	107653 (41.4%)
Female	152066 (58.6%)
Race/ethnicity:	
White, non-Hispanic	109139 (48.9%)
AIAN, non-Hispanic	4024 (1.8%)
Asian, non-Hispanic	19866 (8.9%)
Black, non-Hispanic	40167 (18%)
Hawaiian/Pacific Islander	1867 (0.8%)
Hispanic, all races	48102 (21.5%)
Multiracial, non-Hispanic	179 (0.1%)
Missing	36375 (14%)
Primary language English	206054 (86.1%)
Missing	20268 (7.8%)
Married/partnered	18411 (20.7%)
Missing	170796 (65.8%)
Household size:	
1	45963 (63.4%)
2	9245 (12.7%)
≥ 3	17313 (23.9%)
Missing	187198 (72.1%)
Veteran	430 (0.6%)
Missing	188457 (72.6%)
High income (> 138FPL)	5408 (2.1%)
TANF Benefits	25545 (12.6%)
Missing	57007 (21.9%)
SSI benefits:	
Mandatory or optional	3682 (3.4%)
Not applicable	104153 (96.6%)
Missing	151884 (58.5%)
Psychiatric conditions & counseling:	
ADHD	3052 (1.2%)
Anxiety	26260 (10.1%)
Bipolar	6188 (2.4%)
Depression	20525 (7.9%)
Other mental illness	10473 (4%)
Mental health counseling	21264 (8.2%)
Alcohol use disorder	8388 (3.2%)
Other drug use disorder (non-opioid)	26831 (10.4%)
Nicotine	22593 (8.5%)
Cannabis	4861 (1.8%)
Sedatives	544 (0.2%)
Cocaine	2174 (0.8%)
Other stimulants	2962 (1.1%)
Hallucinogen	188 (0.07%)
Inhalant	90 (0.03%)
Other psychoactive	3693 (1.4%)
**Healthcare Utilization**	
Emergency department	
0	168307 (64.8%)
1-2	74510 (28.7%)
3+	16902 (6.5%)
Inpatient hospitalizations	
0	253678 (97.7%)
1+	6041 (2.3%)
Outpatient visits	
0	28337 (10.9%)
1-20	209354 (80.6%)
21+	22028 (8.5%)

Table e7: continued

Characteristic	Count (%)
**Treatments (month 1)**	
Acetaminophen	12302 (4.7%)
Anti-inflammatory	127092 (48.9%)
Benzodiazepine	10746 (4.1%)
Chiropractic	25749 (9.9%)
Gabapentinoids	15793 (6.1%)
Intervention	4884 (1.9%)
Muscle relaxant	121222 (46.7%)
Massage therapy	25173 (9.7%)
Physical therapy	45080 (17.4%)
Steroid	40289 (15.5%)
Opioid	52713 (20.3%)
≤ 7 days, ≤ 50 MME	35127 (13.5%)
> 7 days, ≤ 50 MME	11878 (4.6%)
> 50 MME	5708 (2.2%)
Other treatment	4413 (1.7%)
**Outcomes (months 2-13)**	
OUD or overdose by 7 months	2199 (1.1%)
OUD or overdose by 13 months	2569 (1.8%)
**Censoring**	
Uncensored through 7 months	197269 (76%)
Uncensored through 13 months	144631 (55.7%)

Binary values formatted as N (%). Continuous values formatted as median (IQR).

© 2026 Doan LV et al. *JAMA Network Open*.

eFigure 9: Estimated Relative Risk of Incident OUD Over 6 Months of Follow-up: Sensitivity Analysis Redefining the Washout Period to Begin 12 Months Before the Initial Treatment Date. Analysis under hypothetically adding a treatment to initial treatment combination and no disenrollment, versus removing the treatment from initial treatment combination and no disenrollment. Error bars denote the 95% CI of the estimate.

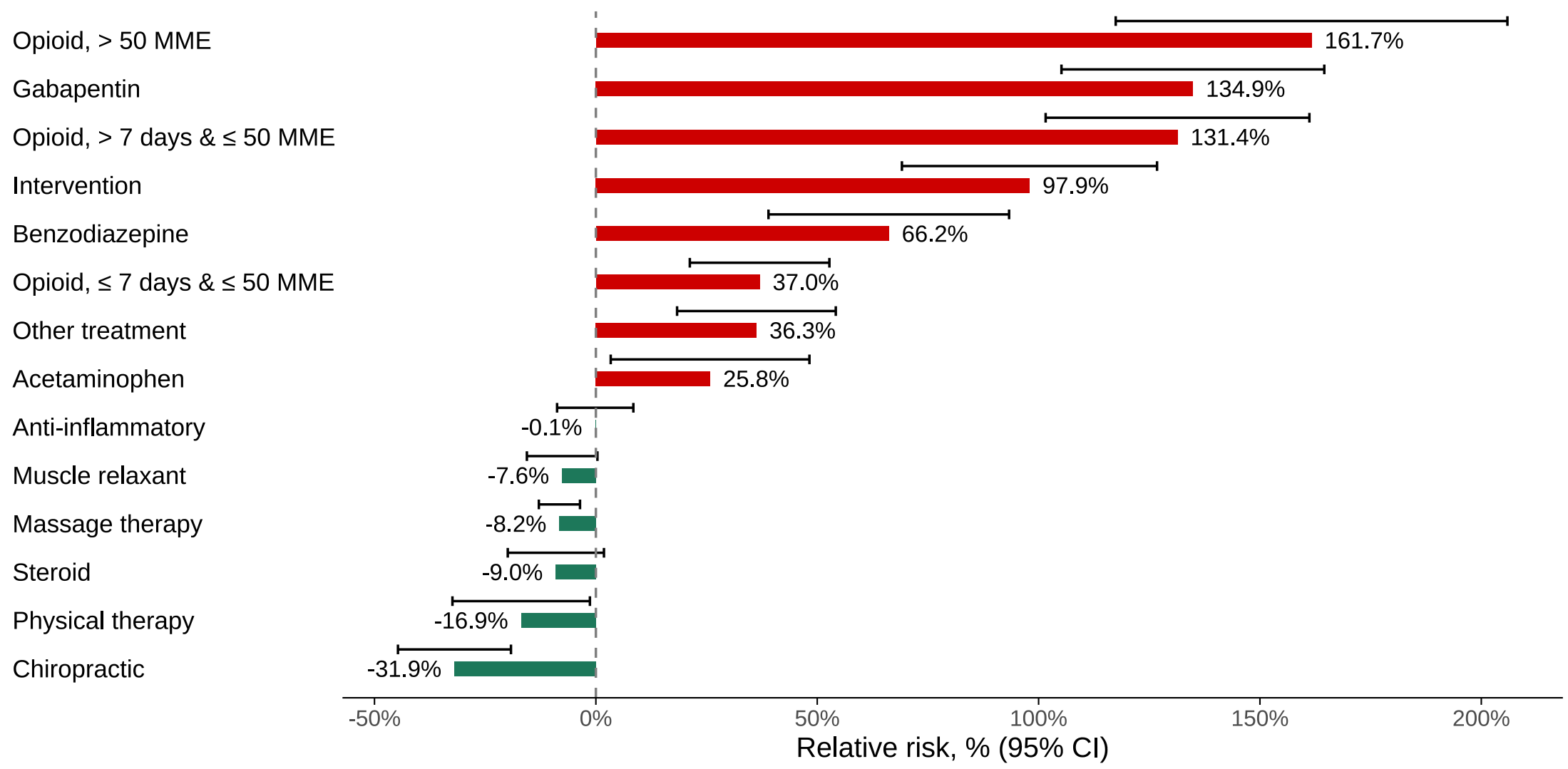

eFigure 10: Estimated Relative Risk of Incident OUD Over 12 Months of Follow-up: Sensitivity Analysis Redefining the Washout Period to Begin 12 Months Before the Initial Treatment Date. Analysis under hypothetically adding a treatment to initial treatment combination and no disenrollment, versus removing the treatment from initial treatment combination and no disenrollment. Error bars denote the 95% CI of the estimate.

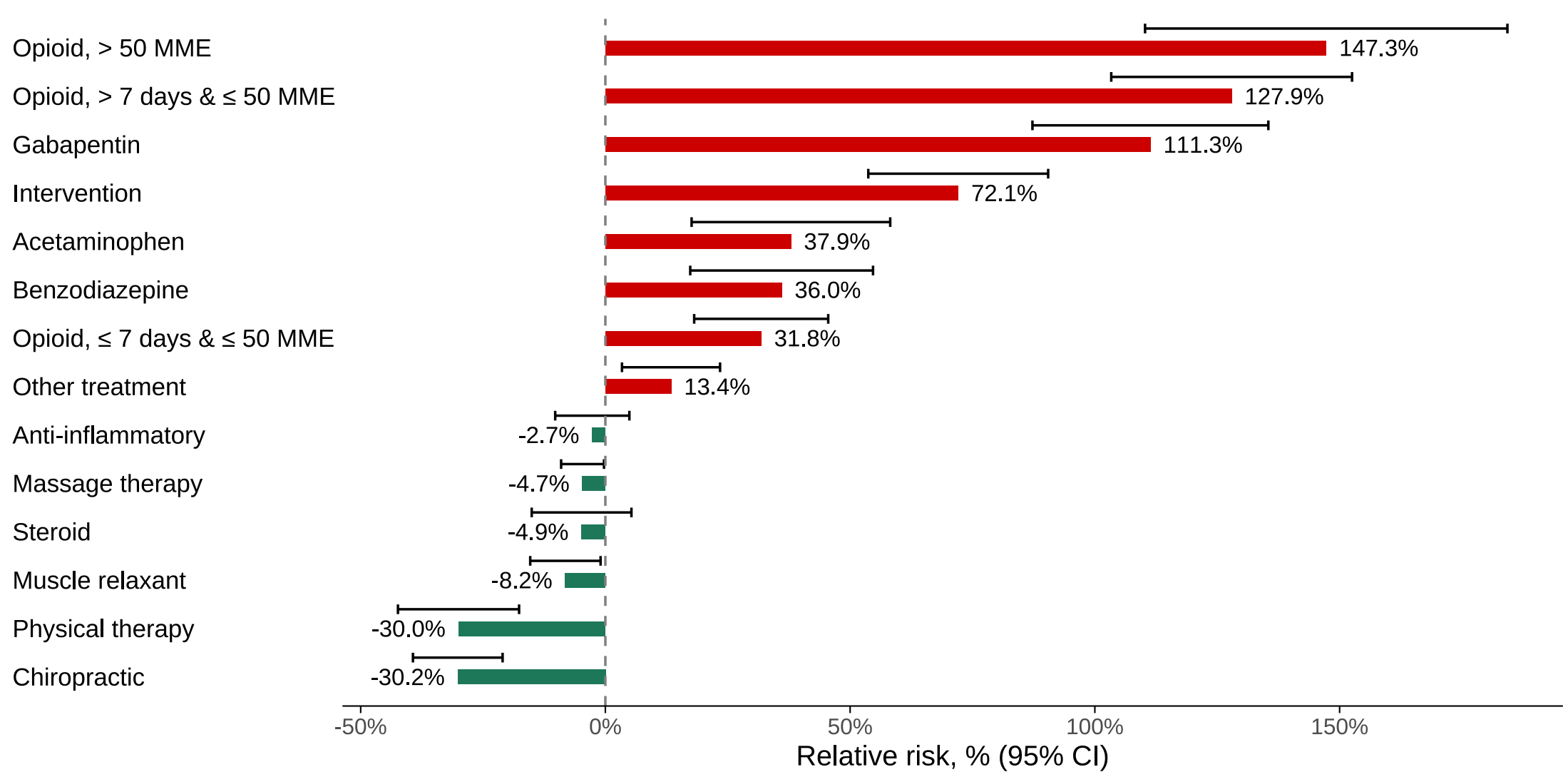

© 2026 Doan LV et al. *JAMA Network Open*.

eReferences

[1] Medicaid Data Quality Atlas `https://www.medicaid.gov/dq-atlas/welcome`. Accessed: 2022-11-10.

[2] Williams A. R., Mauro C. M., Huber B., et al. Defining discontinuation for buprenorphine treatment: implications for quality measurement *American Journal of Psychiatry.* 2024;181:457–459.

[3] Pytell J. D., Fojo A. T., Keruly J. C., et al. Measuring time in buprenorphine treatment stages among people with HIV and opioid use disorder by retention definition and its association with cocaine and hazardous alcohol use *Addiction Science & Clinical Practice.* 2023;18:51.

[4] Case A., Deaton A., Stone A. A.. Decoding the mystery of American pain reveals a warning for the future *Proceedings of the National Academy of Sciences.* 2020;117:24785–24789.

© 2026 Doan LV et al. *JAMA Network Open*.
