# Supplementary material for: Initial Treatment for Acute Low Back Pain and Risk of Opioid Use Disorder and Overdose
Source: JAMA Netw Open. 2026 Oct 19;9(10):e2639420. doi: 10.1001/jamanetworkopen.2026.39420 (PMC13681206; doi:10.1001/jamanetworkopen.2026.39420)
Supplement: Supplementary file 2 [file jamanetwopen-e2639420-s002.pdf]

Data Sharing Statement

Doan. Initial Treatment for Acute Low Back Pain and Risk of Opioid Use Disorder and Overdose. *JAMA Netw Open*. Published October 19, 2026.
doi:10.1001/jamanetworkopen.2026.39420

Data
Data available: No

Additional Information
Explanation for why data not available: We use Medicaid claims data; interested parties may apply to Centers for Medicare & Medicaid Services for access.
